# Oct4-dependent FoxC1 activation improves the survival and neovascularization of mesenchymal stem cells under myocardial ischemia

**DOI:** 10.1186/s13287-021-02553-w

**Published:** 2021-08-28

**Authors:** Zhou Ji, Songsheng Chen, Jin Cui, Weiguang Huang, Rui Zhang, Jianrui Wei, Shaoheng Zhang

**Affiliations:** 1 Department of Cardiology, Guangzhou Red Cross Hospital Medical College of Jinan University, 396 Tongfuzhong Road, Haizhu District, Guangzhou, 510220 China; 2 Department of Cardiology, The Third Affiliated Hospital of Jinzhou Medical University, Jinzhou, 121001 Liaoning China

**Keywords:** FoxC1, Mesenchymal stem cells, Niche, Hypoxic, Oct4, Angiogenesis

## Abstract

**Background:**

The administration of mesenchymal stem cells (MSCs) remains the most promising approach for cardiac repair after myocardial infarct (MI). However, their poor survival and potential in the ischemic environment limit their therapeutic efficacy for heart repair after MI. The purpose of this study was to investigate the influence of FoxC1-induced vascular niche on the activation of octamer-binding protein 4 (Oct4) and the fate of MSCs under hypoxic/ischemic conditions.

**Methods:**

Vascular microenvironment/niche was induced by efficient delivery of FoxC1 transfection into hypoxic endothelial cells (ECs) or infarcted hearts. MSCs were cultured or injected into this niche by utilizing an in vitro coculture model and a rat MI model. Survival and neovascularization of MSCs regulated by Oct4 were explored using gene transfer and functional studies.

**Results:**

Here, using gene expression heatmap, we demonstrated that cardiac ECs rapidly upregulated FoxC1 after acute ischemic cardiac injury, contributing to an intrinsic angiogenesis. In vitro, FoxC1 accelerated tube-like structure formation and increased survival of ECs, resulting in inducing a vascular microenvironment. Overexpression of FoxC1 in ECs promoted survival and neovascularization of MSCs under hypoxic coculture. Overexpression of Oct4, a FoxC1 target gene, in MSCs enhanced their mesenchymal-to-endothelial transition (MEndoT) while knockdown of Oct4 by *si*RNA altering vascularization. In a rat MI model, overexpression of FoxC1 in ischemic hearts increased post-infarct vascular density and improved cardiac function. The transplantation of *ad*Oct4-pretreated MSCs into these ischemic niches augments MEndoT, enhanced vascularity, and further improved cardiac function. Consistently, these cardioprotective effects of FoxC1 was abrogated when Oct4 was depleted in the MSCs and was mimicked by overexpression of Oct4.

**Conclusions:**

Together, these studies demonstrate that the FoxC1/Oct4 axis is an essential aspect for survival and neovascularization of MSCs in the ischemic conditions and represents a potential therapeutic target for enhancing cardiac repair.

**Supplementary Information:**

The online version contains supplementary material available at 10.1186/s13287-021-02553-w.

## Background

Mesenchymal stem cells (MSCs) are multipotent, have low immunogenicity, and are easily obtainable. MSCs secrete angiogenic factors [20] and can repopulate the injured myocardium and restore cardiac function [2, 33]. However, ensuring their long-term survival and viability after transplantation remains a challenge [35]. Animal experiments have shown that MSCs’ survival is foreshortened by the ischemic conditions in the infarcted myocardium [36]. Hypoxia represents one of the stress conditions that can affect MSC survival [21]. Hypoxia negatively influences MSC features, including cell viability, proliferation capacity, differentiation, migration patterns, and metabolism [15]. Thus, new strategies are needed to maintain MSC survival and resist hypoxic injury.

Stem cells are maintained by special microenvironments termed niches. Forkhead box C1 (FoxC1), a member of subfamily FOXC of the forkhead/winged-helix transcription factor (FOX) family, may play an essential role in maintaining the hair follicle stem cell niche that regulates stem cells to preserve their long-term tissue-regenerating potential [25]. Activated FoxC1 regulated the proliferation and self-renewal of arachnoid-pia stem cells in a brain ischemia/reperfusion model by restoring the neuroglial microenvironment [26]. Previously, we identified an endogenous vascular niche that is essential for delaying apoptosis and enhancing stem cells’ regenerative properties when engrafted into ischemic hearts [48, 52]. However, the molecular mechanisms of FoxC1’s effects on MSC function and survival in the ischemic niche are unknown.

In non-small cell lung cancer, FoxC1 knockdown suppressed cancer stem cell self-renewal by decreasing the expression of transcription factor octamer-binding protein 4 (Oct4) and other stemness-related genes [5]. Oct4, encoded by the *Pou5f1* gene, is a member of the POU family. Oct4 is expressed and activated preferentially in embryonic stem cells (ESCs), protecting ESCs against apoptosis and promoting survival [17]. It had been hypothesized that Oct4 acts as a gatekeeper in the beginning of mammalian development and is believed to be permanently epigenetically silenced in adult somatic cells [11]. However, studies have reported Oct4 expression in various stem and progenitor cell populations [27]. Oct4 is also expressed in murine and human adipose-derived stem cells, but decreases after multiple passages, presumably because of disruption of the stem cell niche [16]. Our previous study indicates that Oct4 overexpression enhanced the survival and functions of very small embryonic-like MSCs in infarcted hearts [53]. Importantly, we demonstrated that Oct4 directly functions in MSCs. Therefore, it is important to reassess the interrelationship between FoxC1 and Oct4 and their contribution to the survival and function of stem cells in hypoxic microenvironments. Here, we revealed that Oct4 promotes MSCs to undergo mesenchymal–endothelial transition (MEndoT) in FoxC1-mediated hypoxic microenvironments. We further validate whether FoxC1 targets Oct4 to sustain the survival and function of MSCs.

## Materials and methods

An expanded Materials and Methods section containing details regarding the isolation, culture, and purification of ECs and MSCs; the establishment of a hypoxic coculture model of MSCs and ECs; cell treatments and groups; analysis of cell proliferation and apoptosis; enzyme-linked immunosorbent assays (ELISA); quantitative real-time reverse transcription-PCR (qRT-PCR); immunoblotting; immunocytofluorescence; animal allocation, animal model, study design, gene transfer, cell transplantion, echocardioraphy; and statistics is available in Additional file 9.

### Antibodies and reagents

Additional file 7: Table S1 and Additional file 8: Table S2 list the primer sequences and the antibodies used to analyze mRNA and protein levels, respectively. 4′,6-diamidino-2-phenylindole (DAPI, catalog 28718-90-3) was purchased from Sigma-Aldrich (St. Louis, MO, USA).

### Induction of MI model

Inbred Lewis rats were used. The Animal Care and Use Committee of GuangZhou Red Cross Hospital Medical College of Ji-Nan University approved all animal experiments, which were in compliance with the Guide for the Care and Use of Laboratory Animals published by The National Academies Press (http://www.nap.edu/). Myocardial infarct (MI) were induced in the rats by ligating the left anterior descending coronary artery. Animals with an ejection fraction (EF) < 70% and fractional shortening (FS) < 35% evaluated by echocardiography after induction of MI were selected. Transthoracic Doppler echocardiographic studies were performed with a 7.5-MHz phased-array transducer (Acuson Sequoia 256).

### Histology

The infarct size was determined by calculating the percentage of the infarcted area against the whole LV area using Image J software (Image J 1.52u, http://rsb.info.nih.gov/ij/download/). The peri-infarct regions from the MI model rats and cell therapy rats were embedded in paraffin, sectioned, and stained with triphenyltetrazolium chloride (TTC), hematoxylin and eosin (H&E), and Masson’s trichrome, or by immunohistochemistry (IHC) or immunofluoroscence. MEndoT percentages were derived by counting the number of dually labeled cells and dividing by the number of CD31/EGFP double-positive cells/EGFP-labeled MSCs. The quantitation of vascular area was performed using the JACoP Image J plugin. FoxC1 expression levels in ECs or ischemic hearts were determined using the JACoP Image J plugin [42]. In each case, 5 independent images from each area were analyzed from each section.

### Enzyme-linked immunosorbent assays (ELISA)

The levels of FoxC1, Oct4, Ang-1, bFGF, HGF, VEGF, IL-6, IL-4, and TGF-β1 in supernatant of heart tissues or cells were measured by ELISA using a duoset methodology (R&D Systems; Minneapolis, MN).

### Isolation, expansion, identification, and purification of ECs and MSCs

Primary cultures of microvascular ECs [40] and MSCs [9] were prepared from the left ventricles and bone marrow of adult Lewis rats, respectively, as shown in Additional file 1: Fig. S1. MSCs were isolated and purified from isolated mononuclear cells as described in our previous reports [49, 50]. ECs and MSCs were purified using magnetic-activated cell sorting (MACS).

### Gene expression analyses

Gene expression analyses were performed by Miltenyi Biotec Genomic Services as previously described [56]. The results were viewed as a heatmap using ‘pheatmap’ in the R package [39].

### Gene silencing via RNA interference

For the short hairpin RNA (shRNA) experiments, cells were plated into 10 cm^2^ diameter dishes at a density of 5 × 10^4^ per cm^2^ culture and infected with Lenti-*FoxC1* shRNA (TL513221V, OriGene Technologies), Lenti-*HIF1* shRNA (sc-45919-V, Santa Cruz Biotechnology), Lenti-*cFos* shRNA (sc-29221-SH, Santa Cruz Biotechnology), Lenti-*Bclaf1* shRNA (RC224372L1V, OriGene Technologies), Lenti-*Sp1* shRNA (C02001-7998, GenePharma), Lenti-*Ang1* shRNA (TR711949, OriGene Technologies), Lenti-*bFGF* shRNA (TR710311, OriGene Technologies), Lenti-*HGF* shRNA (TR709834, OriGene Technologies), Lenti-*VEGF* shRNA (TF711624, OriGene Technologies), and Lenti-*FoxO1* shRNA, respectively. The siRNA for *FoxO1* was purchased from Dharmacon (cat. no.D003006060020, Dharmacon Inc, Shanghai, China).

### Assay of cell colony, viability, growth, proliferation, apoptosis, and angiogenesis

Cell colonies were counted visually under an inverted microscope. Cell viability was assessed by visual cell counts after performing a trypan blue exclusion assay. Cell growth was measured using a cell counting kit-8 (CCK-8, Sigma) according to the manufacturer’s protocol. Cell proliferation was assessed using fluorescence staining and FACS for proliferation markers (5-bromodeoxyuridine (BrdU) and Ki67). Cell proliferation index was assessed as the percentage of BrdU-positive nuclei to the total number of nuclei and determined by FACS in the different time of 12 h, 24 h, 36 h, and 48 h after different treatments. Apoptotic cell death under hypoxic conditions was evaluated using annexin V staining and the terminal deoxynulceotidyl transferase nick-end-labeling (TUNEL) assay after 48 h of treatment. Angiogenesis was detected using immunofluroscence with factor VIII positive- staining after 48 h of treatment.

### Quantitative real-time reverse transcription-PCR

Total mRNA was extracted according to standard protocols provided by Invitrogen which served as a template for reverse transcription (RT)-PCR, following standard protocols from Promega. cDNA levels representing gene expression were calculated using quantitative real-time PCR. The primer sequences used are shown in Additional file 7: Table S1.

### Western blotting

Cells or hearts were collected and pulverized to extract protein for immunoblotting. Additional file 8: Table S2 lists the antibodies used to analyze the levels of the transcription factors or cytokines.

### Culture of ECs under hypoxic or normoxic conditions

ECs at passages 2 to 4 were harvested and plated them into 12-well plates (5 × 10^4^ cells/0.5 mL well) with EC medium. Under normoxic or hypoxic conditions, ECs were cultured for analysis of their FoxC1 expression and the effects of FoxC1 on their proliferation and angiogenesis under normoxic condition (NO, serving as the control group) with transfection of *ad*FoxC1 (HO + *ad*FoxC1) or *si*FoxC1 (NO + *s*iFoxC1), and hypoxic culture (HO) with transfection of *ad*FoxC1 (HO + *ad*FoxC1) or *si*FoxC1 (HO + *si*FoxC1). All ECs were cultured for 48 h. After treatments, the cells were collected to analyze proliferation and angiogenesis of ECs.

### Tube formation assay

Liquid Matrigel (BD Biosciences, USA, BD Matrigel Matrix Cat. No. 356234) was added into 96-well tissue culture plates after the induction of EC differentiation into blood vascular cells. ECs (2 × 10^5^ cells per well) were grown in a final volume of 0.30 mL culture medium containing 150 mL M199 (GibcoBRL) and 150 mL CM. Each well was digitally photographed under a phase contrast microscope (Leica). The observed tubes and branching points were counted [44]. Four representative fields are counted and the average of the total area of complete tubes formed by cells per unit area is compared by Image-Pro Plus^®^.

### Acetylated low-density lipoprotein (acLDL) uptake and *Ulex europaeus* agglutinin-1 (UEA-1) binding test

After the induction of MSC differentiation into blood vascular cells, the cells were characterized as adherent cells that were double-positive for DiI-acLDL (Biomedical Technologies, USA) uptake and fluorescein isothiocyanate (FITC)-UEA-1 (Sigma) binding. Cell nuclei were counterstained using DAPI [51].

### Fluorescence-activated cell sorting (FACS) analysis of MSCs

The characteristics of the MSCs were confirmed using FACS with antibodies recognizing CD34, CD44, CD71, CD90, CD147, SH2, SH3, CD45, and CD133 (Additional file 2: Fig. S2).

### Plasmid constructs and transfection

To manipulate the expression of FoxC1/Oct4, an adenoviral vector system was employed. The oligonucleotides and their complementary versions were synthesized by USEN Tech (Shanghai, China), annealed, and ligated into the vectors. *ad*FoxC1/*ad*Oct4 or FoxC1/Oct4 small interfering RNAs (*si*RNAs) and control *si*RNA duplexes were transfected together with the rAAV plasmid vector into ECs/MSCs according to the manufacturer’s protocol, respectively.

### Establishing a hypoxic coculture model of ECs and MSCs

We used Millicell Culture Plate Inserts to establish the hypoxic coculture model of ECs and MSCs. Hypoxic culture was carried out in a humidified, temperature-controlled hypoxia chamber (Coy Laboratories) at 37 ℃, 93% N_2_, 5% CO_2_, and 2% O_2_. ECs (5 × 10^4^ cells/0.5 mL well) were plated in triplicate in 24-well plates and pre-cultured overnight in DMEM. The medium was removed and cocultured with MSCs alone for 108 h under hypoxic conditions (2% O_2_). MSCs and ECs were mixed at a 1:3 ratio in DMEM/medium 199 (M199) and plated onto laminin-coated coverslips as described previously [43]. MSCs were plated onto the upper layer of the cell culture inserts, and ECs were seeded into 35-mm dishes at the bottom of the culture inserts. Polycarbonate membranes (0.2-μm pores; Millipore, Milan, Italy) were located between the two layers, which could enable intercellular signaling while preventing physical contact between the two cell types. Hypoxic cultures were made in a two-gas incubator (Thermo Scientific, Forma™ Steri-Cycle i160 STERI-cycle, USA) equipped with an O_2_ probe to regulate N2 levels. We also set a control group without ECs as feeder layers, with the medium and culture conditions as described above. The cell number was accurately calculated with a cell counter in each time period, and the cell growth curves were drawn.

In the coculture part, ECs were further analyzed by hypoxic coculture for the effects of Oct4 on survival and MEndoT of MSCs. Coculture was performed on MSCs transfected with *ad*Oct4 or *si*Oct4 in the combination with or without transfection of *ad*FoxC1 or *si*FoxC1 in ECs.

### In vitro vascularization of MSCs

MSCs underwent directed differentiation toward blood vascular cells using growth factor supplementation and growth on defined matrices. For vascular differentiation, growth factors bFGF (5 ng/mL; Invitrogen), and VEGF (20 ng/mL; R&D Systems; Minneapolis, MN, USA) were added. The capillary-like structure was viewed 6 h later. Microscopic fields containing the tube structure that formed on the gel were photographed using fluorescence-inverted phase-contrast microscopy. Five fields per test condition were examined [6].

### Angiogenesis assay

The effects on angiogenesis of paracrines secreted by MSCs under hypoxia were studied using ECs + MSCs coculture. The MSCs were lysed with 2 ×  cell lysis buffer (RayBiotech, GA, USA) and quantified using an Angiogenesis Protein Array kit (QAR-ANG-100, RayBiotech).

### EGFP labelling

At 24 h after transfection with the *ad*Oct4, *si*Oct4, or control *si*RNA vectors, cells were co-transfected with a lentiviral vector containing enhanced GFP (EGFP) cDNA, as described previously [24]. More than 70% of MSCs were EGFP-positive, as determined by flow cytometry.

### Rat heart vascular niche model, cell therapy, and groups

After establishment of MI model, the animals were randomly divided into three groups corresponding to FoxC1 transfection status: knockdown of FoxC1 by transfecting the IHs with vectors encoding FoxC1* si*RNA (*si*FoxC1(+) group), no-intervention, and overexpression of FoxC1 by transfecting the IHs with vectors encoding FoxC1 (*ad*FoxC1(+) group). No-intervention rats were transfected with control vectors (−). The rats that were subjected to the same surgical procedure, except for ligation of the coronary artery, received no *ad*FoxC1 or *si*FoxC1, and served as the sham group (Sham). After 15 days post-induction, ten animals in each group were killed to exactly valuate the FoxC1-induced vascular environment at the tissue, cellular, and molecular levels. Meanwhile, other animals received MSCs injection. The animals from the *ad*FoxC1 and control vector groups randomly received injection of MSCs pretreated with *ad*Oct4 or Oct4 small interfering RNAs (siRNAs) and control *si*RNA duplexes.

### Analysis of engraftment and MEndoT of MSCs

Cells were collected from the left ventricles of ten randomly selected hearts per experimental group as previously described [53]. The engraftment was evaluated by determining the number of the expressing EGFP cells per square centimeter in a slice of heart tissue under fluorescence microscope, or expressed as the proportion of cells that expressed EGFP in total cardiomyocytes by FACS. MEndoT percentages were derived by counting the number of dually labeled cells with CD31/EGFP and dividing by the number of EGFP-positive cells.

### Statistical analysis

Data are presented as the mean ± standard error of the mean (SEM). Discrete variables are presented as frequency and proportion. By performing normality test (Shapiro–Wilk test) and homogeneity test of variance, the data that satisfy normal distribution and equal variance assumptions were used for one-way ANOVA analysis of these variables. When the data were conferred for normal distribution but non-homogeneity of variance, Welch ANOVA analyses were performed. Comparisons were performed using the χ^2^ or Fisher’s exact test for discrete variables A 95% confidence interval (CI) (*p* < 0.05) was considered significant.

## Results

### FoxC1 is highly expressed in ischemic ECs

**Fig. 1:**
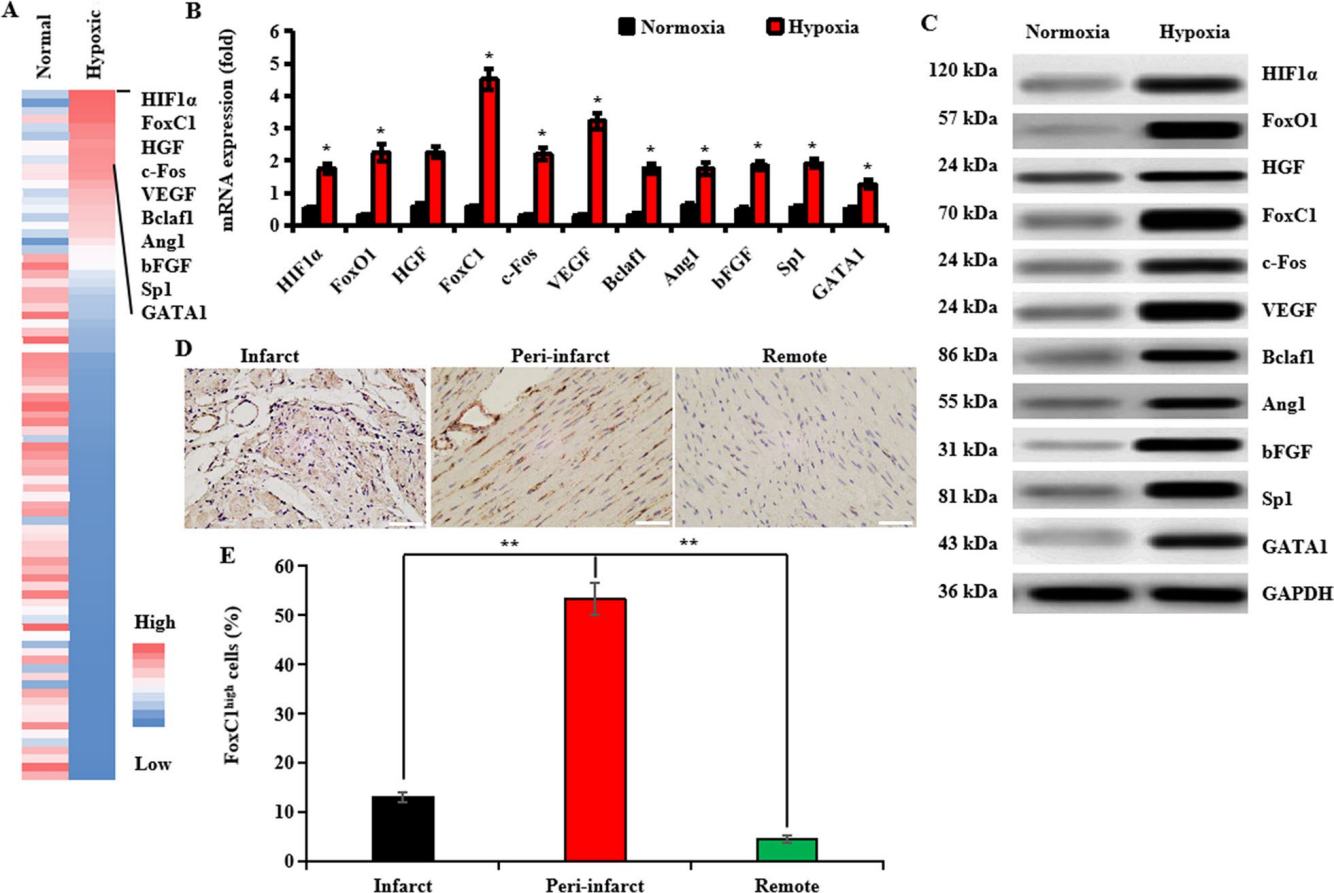
FoxC1 is highly expressed in the ischemic ECs. **A** Heatmap of gene expression levels of 84 TFs. The top 10 highly expressed TFs are listed. Downregulated genes are represented in blue, and upregulated genes are represented in red. **B**, **C** The mRNA and protein expression levels of these TFs were verified in both cultured ECs using qRT-PCR (**B**) and immunoblotting (**C**). All data are the means ± SEM. *p* < 0.05: *versus Normoxia (*n* = 10 per group), One-way ANOVA analysis was used for statistical analysis. **D**, **E** Infarcted heart tissues were stained for immunohistochemistry. Representative images are shown in the infarct, peri-infarct, and remote areas (**D**), and the statistical ratios of FoxC1 highly expressing cells are shown in **E**. FoxC1 was positively stained as brown in the infarcted hearts, and highly seen in the cytoplasm of blood vascular cells around the peri-infarct areas. Scale bars: 50 µm. All Data are shown as means ± SEM. **p < 0.05 (*n* = 10 per group), Welch ANOVA analyses were performed for statistical analysis

We previously demonstrated that the existence of an intrinsic vascular niche appears more beneficial to cardiac repair induced by stem cell therapy after MI, and blood vascular ECs were key cell components in the ischemic niches [52]. von Willebrand factor (vWF, also known as factor VIII) and CD31 are widely used as blood vascular EC markers [52]. We analyzed the expression time curve of blood vessel density in the ischemic areas from the infarcted hearts. Blood vessel density was assessed by anti-factor VIII staining, which showed that blood vessel density gradually increased over time, peaked at 14 days after infarction, and then decreased (Additional file 3: Fig. S3A, B). This observation was confirmed by the occurrence of the highest expressions of angiogenic growth factors Ang1, bFGF, and VEGF in infarcted hearts at this time point (Additional file 3: Fig. S3C–E), suggesting that blood vascular endothelial cells in ischemic heart itself might induce a short-time angiogenesis through activating pro-angiogenic cytokines. Here, we isolated and purified blood vascular ECs from the left ventricles of adult Lewis rats after 14 d of MI with immunomagnetic beads using monoclonal antibodies against vWF and CD31, surface markers of ECs. Normal hearts served as the control. Then, we wanted to identify which transcription factors (TFs) were involved in the maintenance of blood vascular ECs self-renewal. We analyzed TFs based on gene expression profiles of hypoxic ECs and normal ECs derived from infarcted hearts and normal hearts. We chose top-10 TFs that were highly expressed in hypoxic ECs compared with normal ECs (Fig. 1A). qRT-PCR and western blotting detection of the mRNA and protein levels of these TFs in ECs under normoxic or hypoxic culture showed that FoxC1 was most highly expressed in ECs under hypoxia (Fig. 1B, C). Moreover, elevated FoxC1 expression was further confirmed by IHC staining. These expression patterns were seen with the greatest significance in the peri-infarct with a rich vasculature (Fig. 1D, E).

Then, we identified whether FoxC1 was the key transcription factor governing the self-renewal of ECs under hypoxia. We knocked down these selected TFs and performed in vitro clone-formation assays. ECs were cultured for 10 days under hypoxic or normoxic clonal culture conditions (wells > 100 mm^2^ for a 10-day culture period) [1]. Among these 10 TFs, FoxC1 depletion displayed the strongest inhibitory effect on clone-formation (Additional file 4: Fig. S4A, B). Altogether, FoxC1 is highly expressed in the ischemic vascular niche, playing a critical role in the maintenance of myocardial ECs self-renewal. Thereafter, we investigate the effects of FoxC1 on the intrinsic angiogenesis of vascular ECs under hypoxic conditions.

### FoxC1-induced vascular microenvironment after hypoxic endothelial injury

**Fig. 2:**
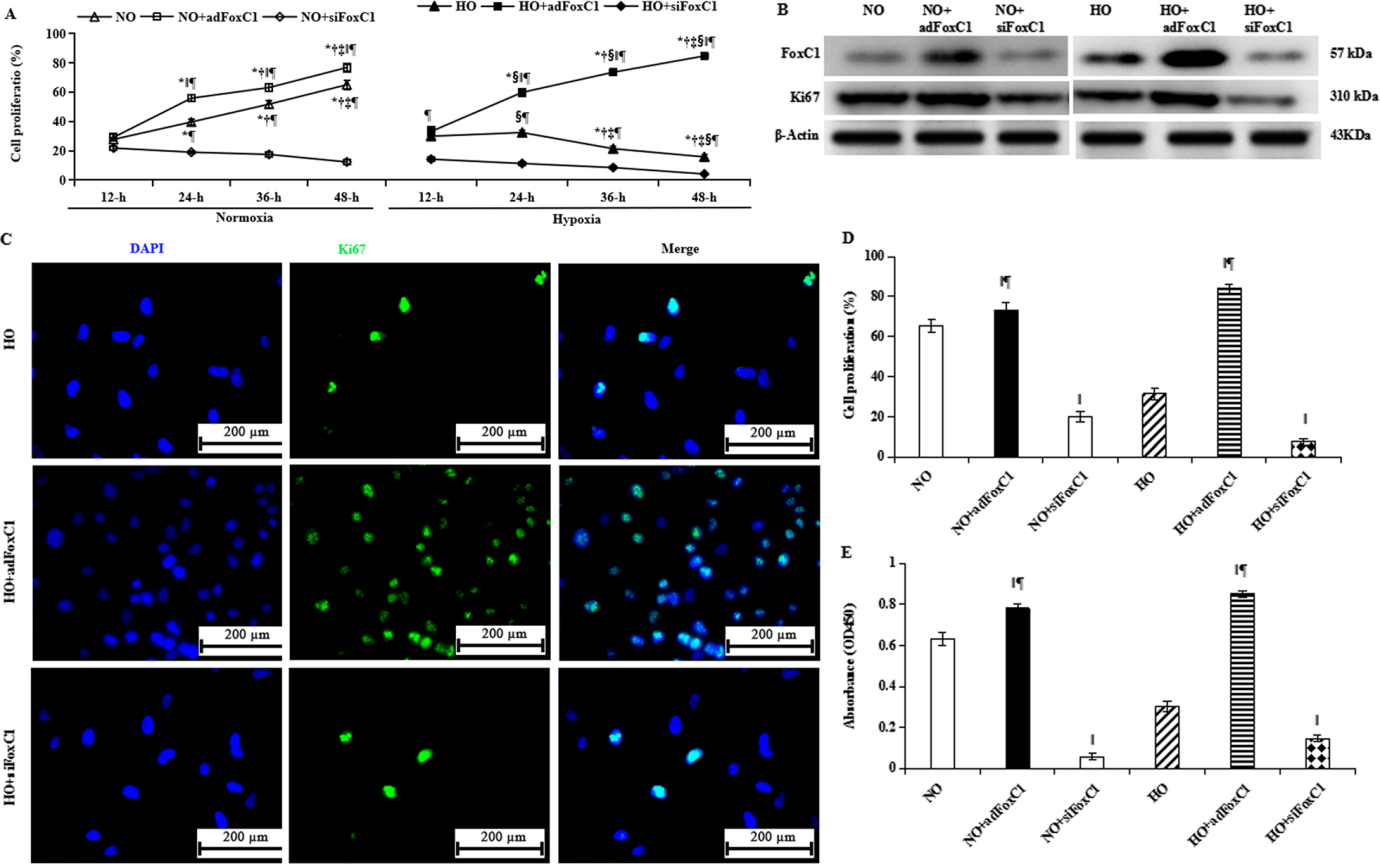
FoxC1 overexpression induces hypoxic EC proliferation. **A** Dynamic analysis of cell proliferation index assessed as the percentage of BrdU-positive cells to total number of cells was determined by FACS in the individual groups at 12-h, 24-h, 36-h, and 48-h after different treatments. **B** Equal protein loading was assessed by immunoblotting for FoxC1, and proliferation marker protein Ki67 at 48 h after treatment. Successful overexpression of the FoxC1 protein was verified by immunoblot analysis in the HO + adFoxC1 group. Note that FoxC1 overexpression strongly induced the expression of Ki67 in this group, while significant decreases in the levels of these proteins were observed in the HO + *si*FoxC1 group. **C** Fluorescence microscope images of the cells double stained with DAPI and Ki67, showing the greatest positive staining in the HO + *ad*FoxC1 group, and the lowest in the HO + *si*FoxC1 group. Scale bars: 200 µm. **D** A percentage of Ki67 positively stained cells was expressed a proliferative index. **E** The proliferative capacities of ECs were also evaluated by CCK-8 assay in optical density (OD) value. Note that all these proliferative indexes of ECs showed the greatest levels in the HO + *ad*FoxC1 group, the second highest in the NO + *ad*FoxC1 group, and the smallest in the HO + *si*FoxC1 group. Data are shown as means ± SEM. Welch ANOVA analyses were performed in **A** and **E**, and one-way ANOVA analysis was used in **D**. *p* < 0.05: *versus at 12-h post-treatment, ^†^versus 24-h post-treatment, ^‡^versus 36-h post-treatment, ^§^versus the normoxia, ^‖^versus the NO/HO group under the same culture conditions, ^¶^versus the *si*FoxC1 group (*n* = 10, each group)

FoxC1 appears to control pathological angiogenesis by regulating VEGF signaling [23]. Previously, we suggested that the existence of an intrinsic vascular niche contributed greatly to the beneficial effects of stem cell therapy on cardiac repairs after MI [48, 52]. To further determine the pathological role of FoxC1 in hypoxic ECs, we established a hypoxic culture model mimicking the characteristics of this niche with FoxC1 overexpression or inhibition in ECs. ECs were transfected with or without *ad*FoxC1 or *si*FoxC1, and cultured for 48 h under hypoxic conditions. Normoxic cultivation of ECs with or without transfection of *ad*FoxC1 or *si*FoxC1 was also performed to clarify these effects under normoxia. Cell proliferation was assessed with the expressions of its markers (Ki67 and BrdU), and CCK-8 assay by using FACS, western blot, and immunofluoroscence. The cell proliferation index (percent proliferating cells) was defined as the ratio of BrdU-positive cells to total cell number, and determined by FACS at each time point after different treatments. As shown in Fig. 2A, dynamic analysis of the percentage of BrdU-positive cells/total number of cells revealed that *ad*FoxC1 stimulated EC proliferation kinetically, and *si*FoxC1 declined this proliferation. Compared with normoxia, this decline was remarkably observed in the *si*FoxC1 treating hypoxic ECs. Similar to BrdU expression, both immunoblot and immunofluoroscence showed that the level of Ki67 were higher in the HO + *ad*FoxC1 group compared with the NO + *ad*FoxC1 group, and lower in the HO + *si*FoxC1 group than in the NO + *si*FoxC1 group (Fig. 2B–D) at 48 h. This observation was confirmed by CCK-8 assay in optical density (OD) value, which was a quantitative index for the proliferative capacity (Fig. 2E). This proliferation difference was consistent with FoxC1 expression. Representative western blots with anti-FoxC1 antibody showed the greatest protein level in the HO + *ad*FoxC1 group, and the smallest level in the HO + *si*FoxC1 group (Fig. 2B). Altogether, FoxC1 is required for the maintenance of ECs proliferation in a hypoxic environment.

**Fig. 3:**
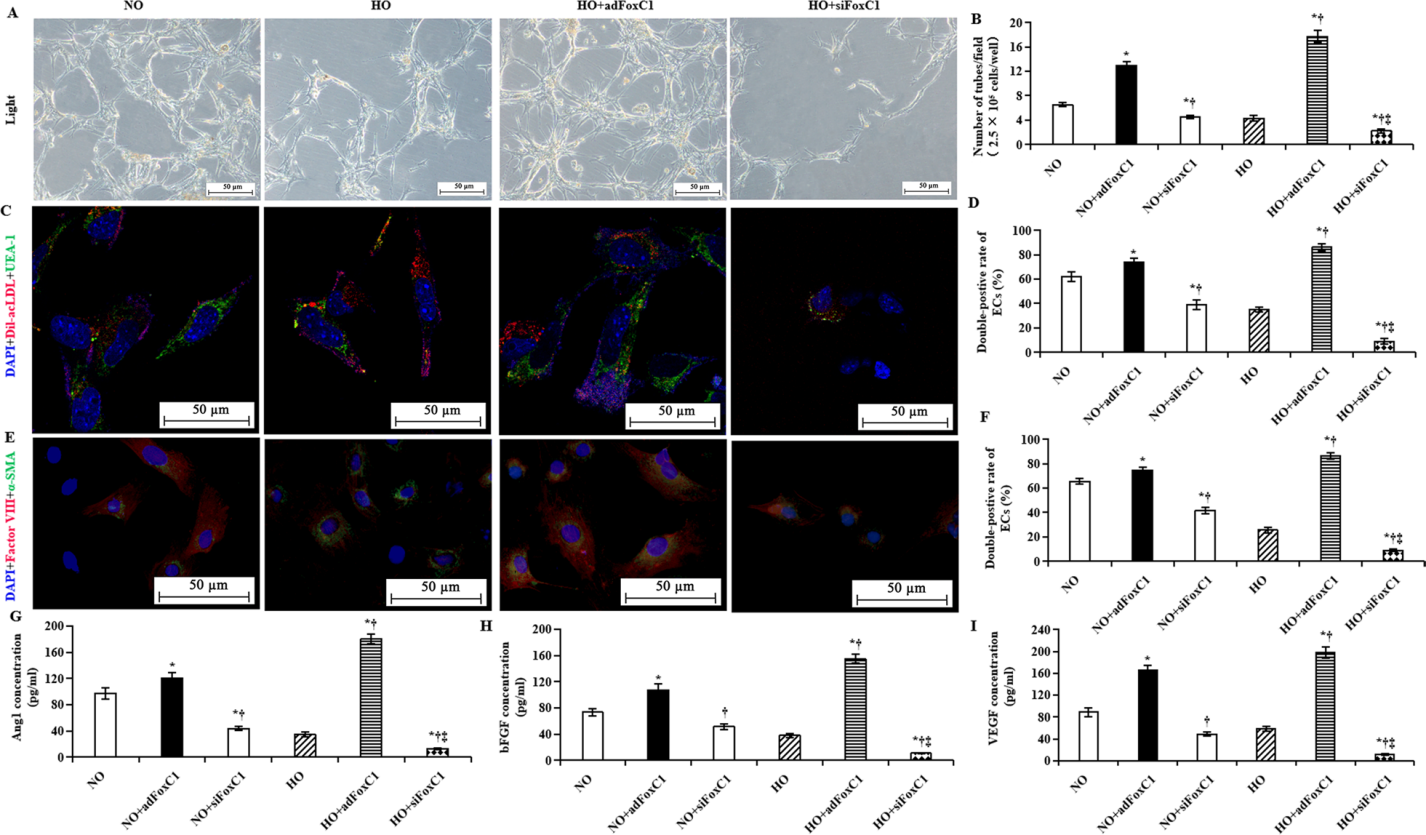
FoxC1-induced the vascular microenvironment under hypoxia. ECs were transfected with FoxC1 vector (*ad*FoxC1), FoxC1 siRNAs (*si*FoxC1), or control siRNA duplexes (Ctrl) and cultured under normoxia or hypoxia. **A** Tube formation of MSCs subjected to hypoxic culture in the absence or presence of *ad*FoxC1, or *si*FoxC1. The number of tube formation was calculated (**B**). **C** Direct fluorescence staining with Dil-acLDL and FITC-UEA-1 in ECs culture system with or without transfection of *ad*FoxC1 or *si*FoxC1, and counterstained with DAPI. **E** Factor VIII and α-SMA expression in MSCs, as determined using immunofluorescence with anti-factor VIII (red) and anti-α-SMA (green) antibodies, and the nuclei were stained blue using DAPI. Scale bars = 50 µm. Double-positive cells in **D** and **F** were expressed as a percentage of Dil-acLDL^+^ UEA-1^+^ DAPI^+^ (**D**) or Factor VIII^+^α-SMA^+^DAPI^+^ (**F**) relative to all DAPI^+^ cells. **G**–**I** ELISA assay of difference in pro-angiogenic factors, Ang1 (**G**), bFGF (**H**), and VEGF (**I**) at 48 h after nomorxic or hypoxic culture in the conditioned culture supernatants of the ECs transfected with FoxC1vector (*ad*FoxC1), FoxC1 siRNAs (*si*FoxC1), or control siRNA duplexes (CON). All data are the means ± SEM. Welch ANOVA analyses were performed in **B**,** G**, **H**, and **I**, and one-way ANOVA analysis was used in **D** and **F**. *p* < 0.05: *versus the NO/HO group under the same culture conditions, ^†^versus the NO + *ad*FoxC1group, ^‡^versus the HO + *ad*FoxC1 group (*n* = 10 per group). HPF, high-power field, acLDL, acetylated low-density lipoprotein, DAPI, 4′, 6-diamidino-2-phenylindole, FITC-UEA fluorescein isothiocyanate-labeled Ulex europoeus agglutinin I lectin

We next observed the promotion of FoxC1 on differentiation of ECs. We observed that FoxC1 overexpression in hypoxic ECs remarkably increased tube formation in the HO + *ad*FoxC1 group, and FoxC1 knockdown significantly decreased tube formation in the HO + *si*FoxC1 group (Fig. 3A). Statistical analysis showed that compared to the HO group, the number of tubes per field increased 1.5-fold in the *ad*FoxC1 group and decreased 74% in the *si*FoxC1 groups (Fig. 3B). We performed immunofluorescent staining for ECs and observed that 86% of ECs exhibited both UEA-1 and acLDL fluorescence in the *ad*FoxC1 group, whereas only 9% of ECs took up UEA-1 and acLDL in the *si*FoxC1 group (Fig. 3C, D). High take-up of UEA-1 and acLDL was in agreement with high expression of the endothelial transcription factors, α-SMA and factor VIII, in *ad*FoxC1-treated ECs. Immunofluorescence showed that the number of ECs with double-positive staining for α-SMA and factor VIII was 2.4-fold and 7.6-fold higher in the HO + *ad*FoxC1 group than in the HO and the HO + *si*FoxC1 groups, respectively (Fig. 3E, F). Similar observations were achieved in the expression of pro-angiogeneic cytokines in hypoxic ECs after FoxC1 intervention. ELISA results showed compared with the cells in the HO group, FoxC1 overexpression significantly promoted expression of vascular growth factors in the HO + *ad*FoxC1 group, such as Ang-1, bFGF, and VEGF: *si*FoxC1 abolished this promotion (Fig. 3G, H, I). However, to our surprise, such changes are not so obvious in normoxic ECs after overexpression or knockdown of FoxC1. Altogether, FoxC1 promoted ECs to establish an intrinsic vascular microenvironment under hypoxia.

Next, we determined whether the FoxC1-induced vascular microenvironment could benefit the survival and angiogenesis of MSCs under hypoxia.

### MSCs adopt an endothelial like fate in FoxC1-induced vascular microenvironments under hypoxia

**Fig. 4:**
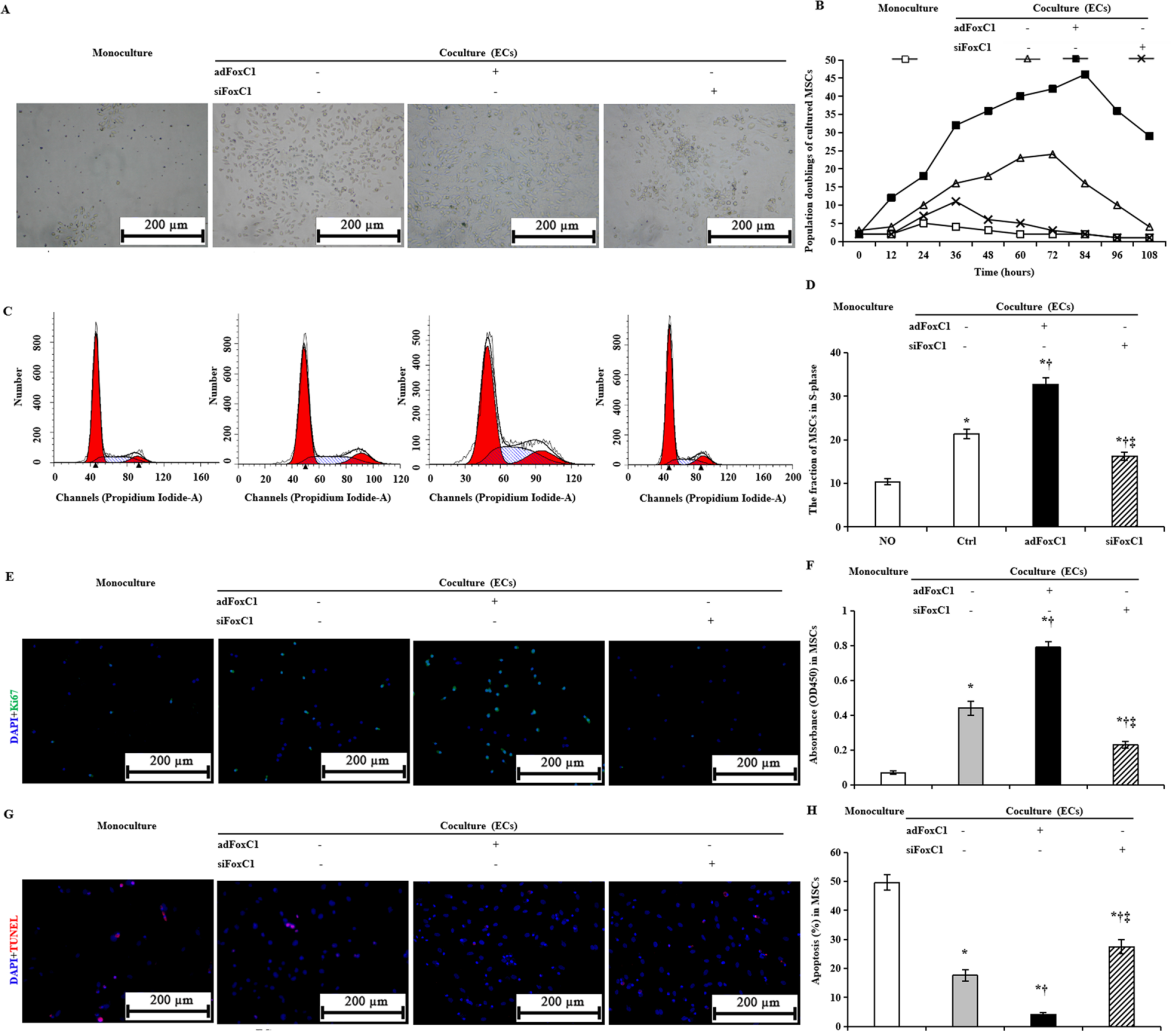
FoxC1 overexpression in ECs promotes self-renewal and survival of MSCs under hypoxia. **A** Representative growth state and morphology of the MSCs without (monoculture) or with coculture with the ECs alone, *ad*FoxC1-treated, and *si*FoxC1-treated ECs in 108 h of culture under hypoxia conditions. Transfection of *ad*FoxC1 improves self-renewal of MSCs, and FoxC1 deficiency impaired this effect. Scale bars = 200 µm. **B** MSCs were cultured for 108 h under hypoxic conditions, and the number of cumulative population doublings was determined. **C** For cell cycle distribution, MSCs without (monoculture) or with coculture with ECs treated with or without *ad*FoxC1 or *si*FoxC1 for 108 h were collected and detected by flow cytometry, the percentage of of MSCs at S phase of the cell cycle was statistically analyzed as a histogram. **D** The percentage of cells in S phase was determined in MSCs without (monoculture) or with coculture with the ECs treated with or without *ad*FoxC1 or *si*FoxC1. **E** MSCs after 108 h of hypoxic culture were stained with anti-Ki67 antibodies and then counterstained with DAPI, followed by confocal microscopy. Scale bars = 200 µm. **F** The proliferation of MSCs cocultured with *ad*FoxC1- treated ECs was remarkably higher than the other MSCs, as assessed using a CCK8 assay. **G** MSCs after 108 h of hypoxic culture were evaluate by using the TUNEL assay. TUNEL-positive cells were obviously decreased in the MSCs cocultured with *ad*FoxC1- treated ECs as compared with the other MSCs. Scale bars = 200 µm. **H** Statistical analysis of the mean percentage of annexin V-positive cells relative to the total number of MSCs. FoxC1 overexpression in ECs reduced the apoptosis of MSCs, and FoxC1 deficiency increased apoptosis. All data are the means ± SEM. One-way ANOVA analysis was used in **D**, and Welch ANOVA analyses were performed in **F** and **H**. *p* < 0.05: *versus monoculture, ^†^versus ECs alone, and ^‡^versus *ad*FoxC1 + ECs (*n* = 10 in each group)

To further determine the effect of FoxC1 in ECs on MSC self-renewal under hypoxia, ECs were transfected with *ad*FoxC1, *si*FoxC1, or control vectors (Ctrl), and cocultured with MSCs. Single-cloned MSCs were expanded and then analyzed by FACS. The results indicated that these cells were positive for the MSC markers SH2, SH3, CD44, CD71, CD90, and CD147, at 97.3%, 97.7%, 95.6%, 97.5%, 96.8%, and 98.1%, respectively; and were negative for hematopoietic markers CD34, CD45, and CD133, at 0.83%, 0.95%, and 1.13%, respectively, which indicate pure MSCs (Additional file 2: Fig. S2). ECs and MSCs were cocultured under hypoxic conditions for 108 h. Monoculture of MSCs served as control in the same conditions. MSCs cocultured with ECs increased the cell doubling upon 72 h of hypoxia exposure when compared with monoculture control, but then the cell doubling gradually decreased. When the cultures were transferred to ECs transfected with *ad*FoxC1, cell doubling increased up to 84 h, while silencing FoxC1 in ECs markedly reduced MSC growth (Fig. 4A, B). Consistent with the cell doubling changes seen after 72 h, S-phase fraction of MSCs increased in coculture condition: compared with monoculture, MSC S-phase fraction increased 1.1-fold in ECs alone and 2.2-fold in ECs transfected with *ad*FoxC1 (Fig. 4C, D). Cell proliferation and growth were also assessed using Ki67 staining and CCK8 assays. Similarly, the MSCs double-positively stained with DAPI and Ki67 were the greatest in the ECs + *ad*FoxC1 group, the secondary in the ECs alone, and the smallest in the monoculture without ECs and FoxC1 (Fig. 4E). After 108 h of hypoxic culture, MSC viability, as assessed using CCK8 assays, was much higher in the ECs + *ad*FoxC1 group than in the other groups (Fig. 4F, 0.79 ± 0.10 in the ECs + *ad*FoxC1 group versus 0.07 ± 0.03 in the monoculture group, versus 0.44 ± 0.11 in the ECs alone group, and versus 0.23 ± 0.06 in the ECs + *si*FoxC1 group, all *p* < 0.001). This suggested that coculture with *ad*FoxC1 treating ECs could increase the proliferation capacity of MSCs under hypoxia significantly. The effect of FoxC1 on MSC apoptosis post-hypoxic culture was evaluated using TUNEL assays and Annexin V-APC and PI staining, followed by flow cytometry. The the proportion of TUNEL^+^ cells (Fig. 4G) and annexin V positive cells (Fig. 4H) were lower in MSCs cocultured with ECs coculture than in MSCs monoculture, dramatically lower in MSCs cocultured with *ad*FoxC1-treated ECs than in MSCs cocultured with ECs alone. However, *si*FoxC1 in ECs eliminated this reduction. Thus, FoxC1 is required to maintain MSC self-renewal and survival under hypoxic conditions.

### FoxC1 initiates activation of Oct4 signaling

**Fig. 5:**
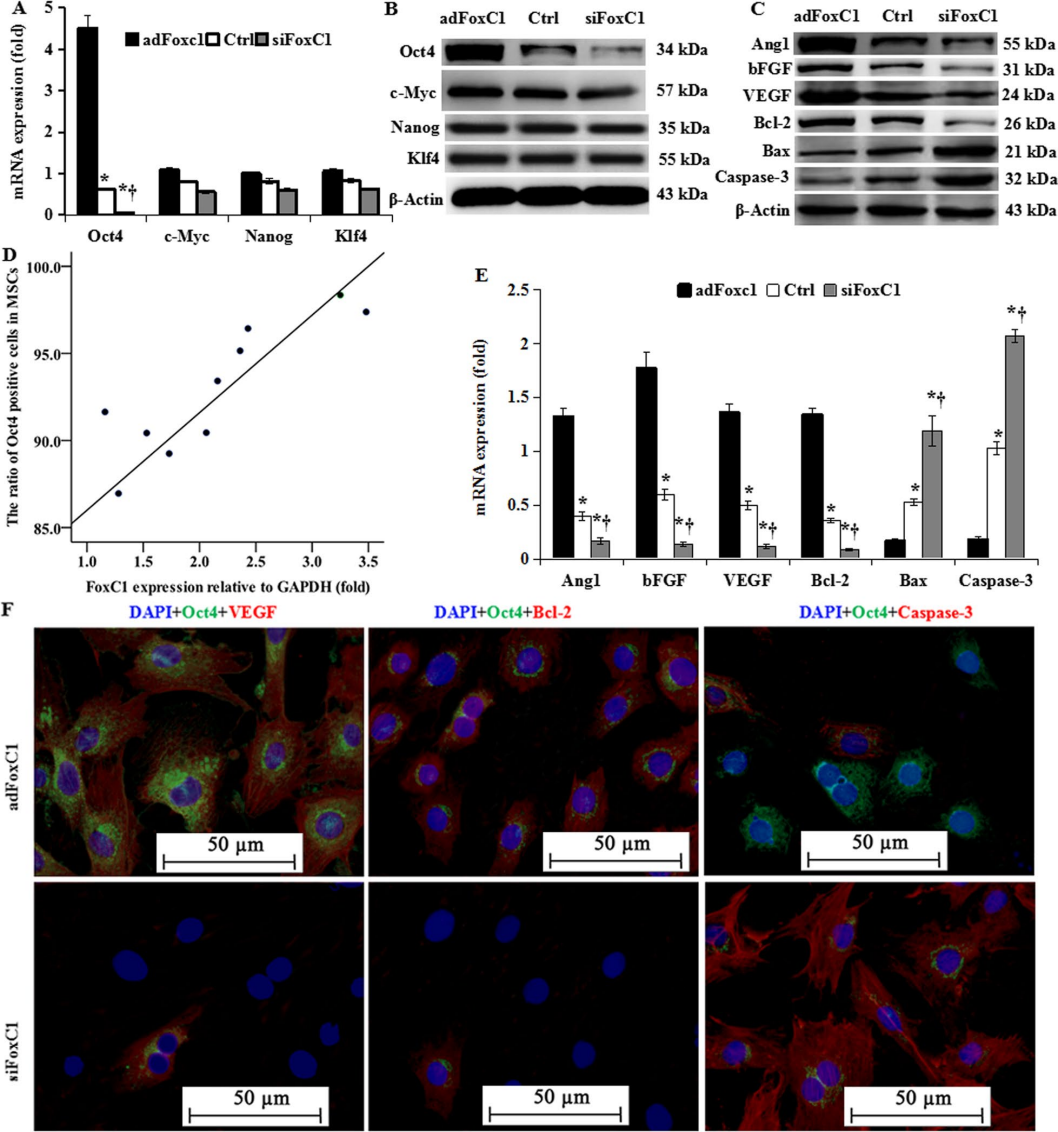
FoxC1 initiates Oct4 activation. **A**, **B** FoxC1 overexpression in ECs increases the expression of Oct4 mRNA and protein in cocultured MSCs. The indicated core stemness factors were analyzed in the MSCs cocultured with *ad*FoxC1-treated ECs, Ctrl-ECs, or *si*FoxC1-treated ECs. **C**, **E** The mRNA and protein expressions of Oct4-relative pro-angiogenic factors Ang1, bFGF and VEGF, anti-apoptic factor Bcl-2, and pro-apoptic factors Bax and caspase3 were detected by immunoblotting (**C**) and real-time RT-PCR (**E**) in these MSCs cocultured with *ad*FoxC1-treated ECs, Ctrl-ECs, or *si*FoxC1-treated ECs. **D** Correlation analysis of Oct4 positive MSCs cocultured with *ad*FoxC1-treated ECs and FoxC1 expression in the ECs transfected with *ad*FoxC1 were analyzed by quantitative immunofluorescence and western blotting. **F** Representative immunostaining in MSCs cocultured with *ad*FoxC1-treated ECs or *si*FoxC1-treated ECs showed Oct4 expression was consistent with VEGF and Bcl-2 expression, but opposite to caspase 3 expression. The nuclei of MSCs were stained blue using DAPI. Scale bars = 50 µm. All data are the means ± SEM. *p* < 0.05: *versus *ad*FoxC1 group, ^†^versus Ctrl group (*n* = 10 per group). Welch ANOVA analyses were performed in **A** and **E**

We next investigated the mechanisms by which FoxC1 regulates MSC survival under hypoxia. Oct4, a homeobox transcription factor is essential for MSC self-renewal [4]. To determine the underlying mechanism of Oct4 in FoxC1-mediated survival of MSCs, we analyzed key TFs related to MSC stemness. We noticed that FoxC1 overexpression in ECs remarkably increased Oct4 mRNA expression in MSCs, whereas Oct4 mRNA was almost undetectable in MSCs cocultured with FoxC1-deficient ECs (Fig. 5A). Moreover, Oct4 protein levels were remarkably reduced in MSCs cocultured with *si*FoxC1-treated ECs (Fig. 5B). However, alteration of FoxC1 expression did not cause a significant change in expression of c-Myc, Nanog, and KLF4 (Fig. 5A, B), suggesting that Oct4 is the primary regulator of these stemness factors, mediated by FoxC1 in MSCs. The proportion of Oct4 positive cells among MSCs correlated positively with FoxC1 protein levels in *ad*FoxC1-treated ECs (*r* = 0.873, *p* = 0.001, Fig. 5D). Taken together, FoxC1 is essential for the activation of Oct4 signaling. Importantly, mRNA and protein levels of Oct4-related angiogenic factors, Ang1, bFGF, and VEGF, and anti-apoptotic protein Bcl-2 were elevated dramatically in MSCs cocultured with *ad*FoxC1-treated ECs compared with that in MSCs cocultured with Ctrl-ECs, whereas depletion of FoxC1 abolished this increase (Fig. 5C, E). Conversely, the influence of FoxC1 on apoptotic signaling molecules, including Bax and caspase3, showed the opposite trend: FoxC1 overexpression down regulated Bax and caspase3 expression, and *si*FoxC1 upregulated their expression. Moreover, immunofluoroscence revealed that Oct4 expression was consistent with the expressions of VEGF and Bcl-2, but opposite to caspase 3 expression (Fig. 5F). These results suggested that ECs induced survival and self-renewal of MSCs under hypoxic conditions via the FoxC1-mediated Oct4 pathway.

### Oct4 promotes MEndoT of MSCs in FoxC1-mediated microenvironments

**Fig. 6:**
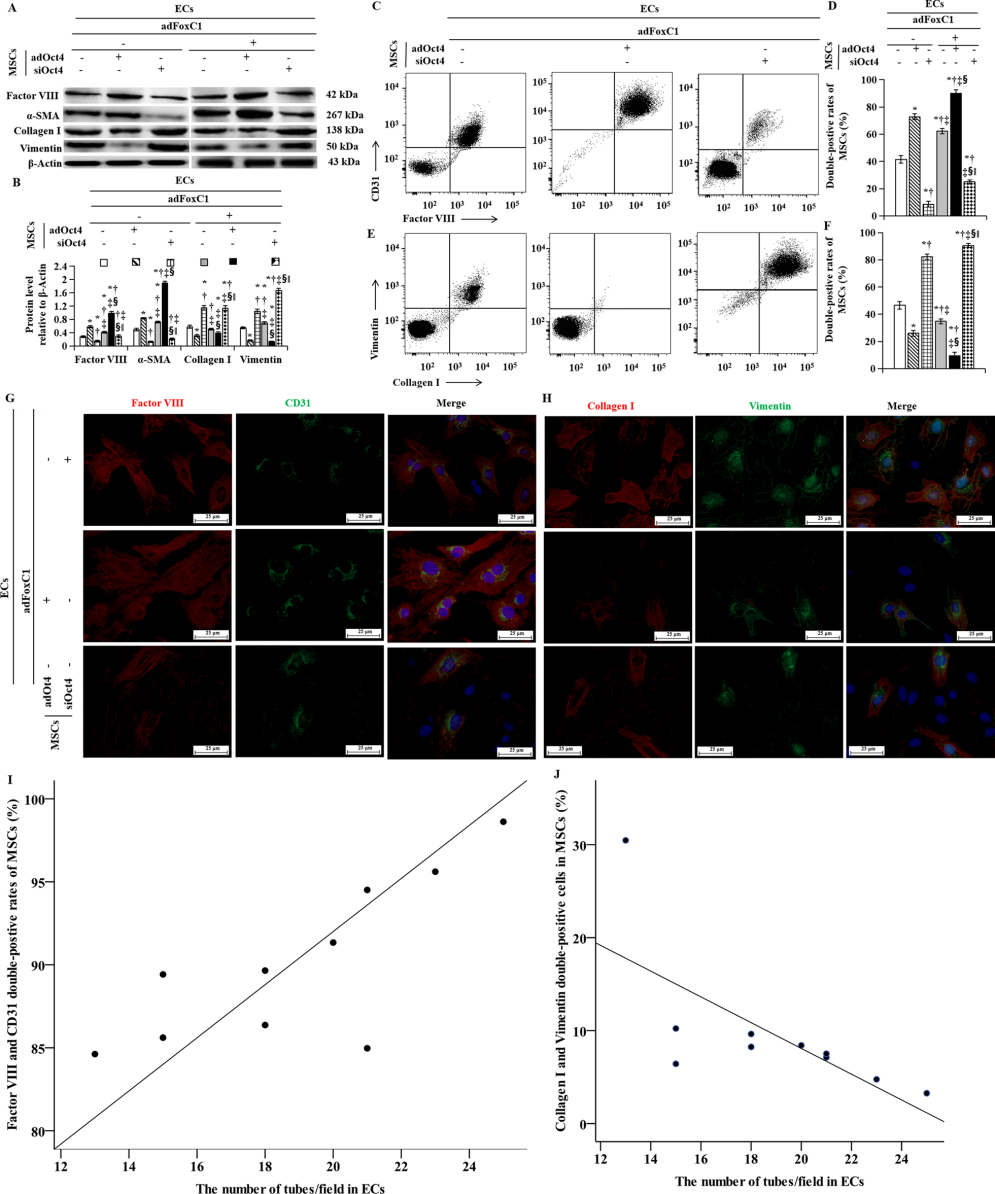
MSCs in FoxC1-mediated vascular microenvironments adopt endothelial cell fates after Oct4 overexpression. **A** Angiogenesis and fibrosis of MSCs subjected to transfection of *ad*Oct4, *si*Oct4, or no intervention in the absence or presence of *ad*FoxC1, as determined using immunoblotting with anti-factor VIII/α-SMA and collagen I/vimentin, respectively. **B** The histogram shows the quantification of the protein level relative to β-actin. **C**–**F** Double-positive cells in angiogenesis or fibrosis proteins were expressed as a percentage of factor VIII^+^CD31^+^ (**C**,** D**) or collagen I^+^vimentin^+^ (**E**, **F**) relative to all MSCs by FACS. **G**, **H** Direct fluorescence staining with VIII/α-SMA and collagen I/vimentin in MSCs culture system with or without transfection of *ad*FoxC1, and counterstained with DAPI. Scale bars = 25 µm. **I**, **J** Correlation analysis of the double-positive rates of VIII/α-SMA (**I**) and collagen I/vimentin (**J**) in MSCs with the number of tubes/field in ECs. All data are the means ± SEM. *p* < 0.05: in ECs without adFoxC1, *versus MSCs treated with no intervention, ^†^versus MSCs with *ad*Oct4, ^‡^versus MSCs with *si*Oct4; in ECs with *ad*FoxC1, ^§^versus MSCs treated with no intervention, ^‖^versus MSCs with *ad*Oct4 (*n* = 10 per group). Welch ANOVA analyses were performed in **B** and **D**, and one-way ANOVA analysis was used in **F**. DAPI, 4′, 6-diamidino-2-phenylindole; FACS, fluorescent-activated cell sorting

The differentiation of MSCs towards adipocyte, osteoblast, and chondrocyte lineages is well established, and MSCs have a clear tendency to organize into clusters and form capillary-like structures [22, 31]. Batlle et al. found that MEndoT of MSCs contributes to cancer cell proliferation in perivascular locations [3]. FoxC1 induces vascular environments and upregulates Oct4 under hypoxia; therefore, we next determined whether Oct4 regulate the MEndoT of MSCs in FoxC1-induced vascular environments. MSCs were transfected with vectors encoding Oct4 (*ad*Oct4), Oct4 *si*RNA (*si*Oct4), or no intervention (−), cocultured with *ad*FoxC1 (+) or control vector (−)-treated ECs, seeded on Matrigel to facilitate angiogenesis, and subjected to serum starvation under hypoxia. After 72 h of hypoxia cocultivation, MSCs were collected, and their angiogenesis and fibroblast were analyzed by western blotting, flow cytometry and immunofluorescence techniques. Western blotting showed the highest expressions of the vascular characteristic markers, factor VIII and α-SMA in the Oct4-transfected MSCs cocultured with the FoxC1 overexpressing ECs, followed by that in the *ad*Oct4-transfected MSCs cocultured with the control vector-treated ECs, and the lowest was observed in the *si*Oct4-transfected MSCs cocultured with the control vector-treated ECs (Fig. 6A, B). Conversely, Oct4 overexpression dramatically decreased the protein levels of fibroblast markers, collagen I and vimentin, in MSCs cocultured with *ad*FoxC1-treated ECs, whereas the levels of these marker proteins were significantly higher in *si*Oct4-treated MSCs cocultured with the control vector-treated ECs. The FACS-evaluated expression of endothelial specific surface markers, such as vWF and CD31, was higher in MSCs treated with *ad*Oct4 than in those treated with Oct4 *si*RNA and no intervention, and was the highest in MSCs treated with both Oct4 transfection and *ad*FoxC1-treated ECs (Fig. 6C, D). Expressions of collagen I and vimentin showed the opposite pattern (Fig. 6E, F). Immunofluoroscence showed similar results after Oct4 overexpression (Fig. 6G, H). Upregulating Oct4 in MSCs increased the expressions of factor VIII and CD31, and reduced the levels of collagen I and vimentin, while downregulating Oct4 showed the opposite results. Correlation analysis showed that the number of tubes/field in ECs was positively correlated with factor VIII and CD31 double-positive rates of MSCs (Fig. 6I, *r* = 0.779, *p* < 0.01), and negatively correlated with collagen I and vimentin double-postiive rates of MSCs (Fig. 6J, *r* =  − 0.693, *p* < 0.05). All these data suggested that Oct4 improved FoxC1-mediated MEndoT of MSCs under hypoxia.

### Oct4 overexpression upregulated angiogenesis-related cytokines in MSCs

**Fig. 7:**
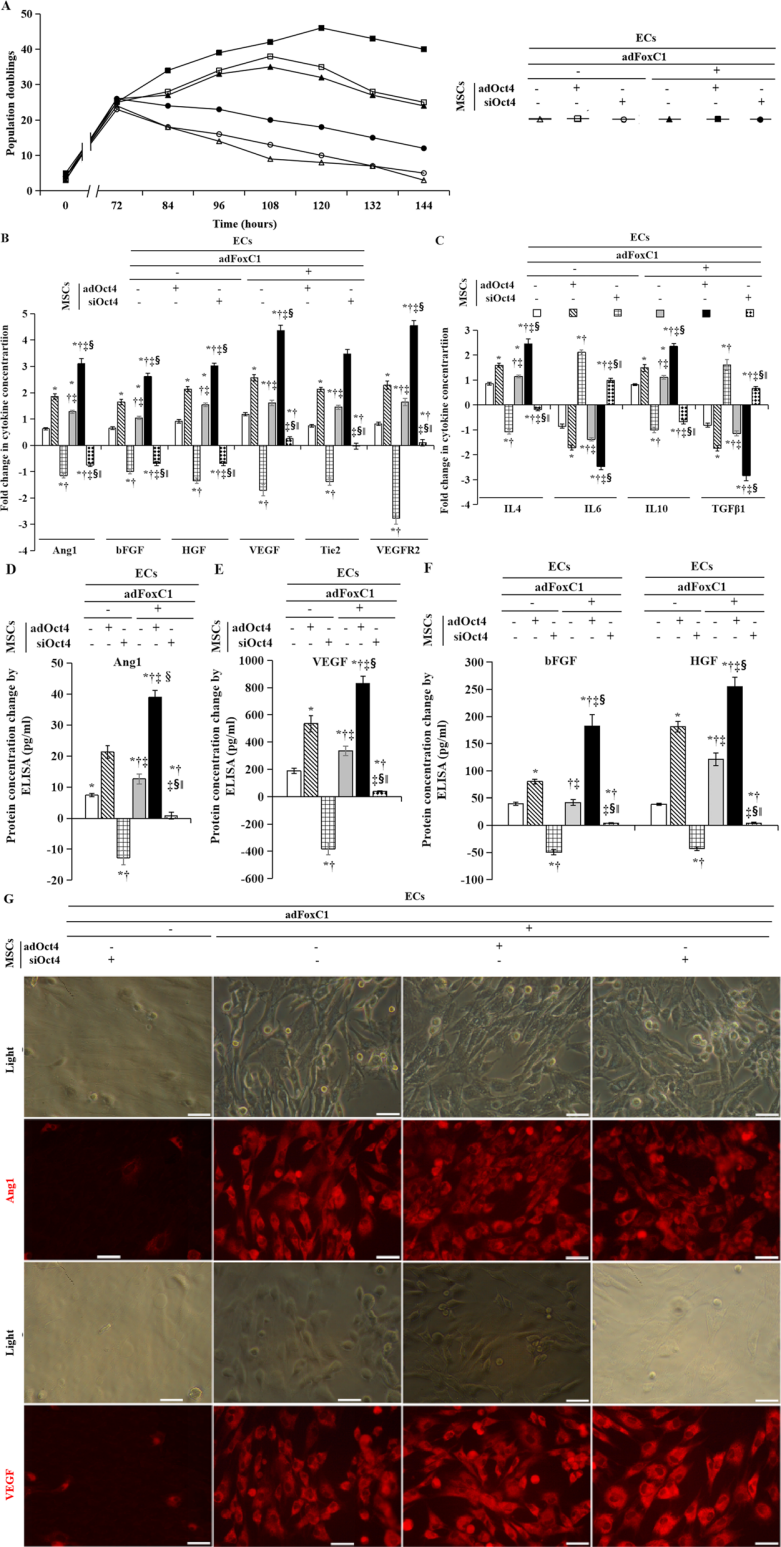
Oct4 improved the expression levels of pro-angiogenic factors in adFoxC1-mediated vascular microenvironments. **A** In growth kinetic analyses, a significantly increasing pattern was seen in MSCs cocultured with *ad*FoxC1-treated ECs after *ad*Oct4 transfection, which also extended the peak cell growth in *ad*Oct4 transfected MSCs cocultured with ECs alone. **B**, **C** Comparison of angiogenic factor concentration changes under hypoxic conditions, as detected using an antibody array in MSCs before and after Oct4 overexpression. **D**, **E**, **F** Quantitative analysis of Ang1 (**D**), VEGF (**E**), and bFGF and HGF (**F**) protein level changes in the conditioned culture supernatants of the MSCs treated with control vector, *ad*Oct4, or *si*Oct4 in the presence or absence of *ad*FoxC1 in ECs. All data are the means ± SEM. *p* < 0.05: in ECs without *ad*FoxC1, *versus MSCs treated with no intervention, ^†^versus MSCs with *ad*Oct4, ^‡^versus MSCs with *si*Oct4; in ECs with *ad*FoxC1, ^§^versus MSCs treated with no intervention, ^‖^versus MSCs with *ad*Oct4 (*n* = 10 per group). Welch ANOVA analyses were performed in **B** and **C**–**F**. **G** Ang1 and VEGF immunocytofluorescence in MSCs 144 h after the imposition of hypoxic conditions. (The first and third rows) High magnification of MSCs under a light microscope. (The second and fourth rows) Immunofluorescence staining for Ang1 (red) and VEGF (red) on MSCs harvested 144 h following hypoxic culture to detect Ang1 and VEGF levels in the cytoplasm of MSCs. Scale bar: 50 μm

We used a rat angiogenesis array to analyze the expression of 60 angiogenesis-related cytokines from MSCs cocultured in *ad*FoxC1-treated ECs, before and after *ad*Oct4 or *si*Oct4 transfection. MSCs peaked at 72 h post-hypoxic coculture with ECs alone and decreased thereafter as mentioned above; therefore, we chose this time point to induce MSCs Oct4 overexpression or knockdown by adding *ad*Oct4, *si*Oct4, or control vector, and coculture these cells with ECs pretreated with *ad*FoxC1 or not. Compared with adding control vector, Oct4 overexpression dramatically increased the growth of MSCs, and extended their cell doubling peak time to 120 h after coculture with adFoxC1-treated ECs. However, *si*Oct4 significantly reduced this proliferation (Fig. 7A). The expression of these angiogenesis-related factors was examined pre-Oct4 treatment (72 h of culture) and at 144 h after treatment of *ad*Oct4, *si*Oct4, or control vector. We compared the changes in the levels of these cytokines before and after Oct4 treatment and found significantly increased levels of Ang1, bFGF, HGF, IL-4, IL-10, Tie2, VEGF, and VEGFR2 in MSCs after Oct4 overexpression, whereas the protein levels of IL-6 and TGFβ1 decreased significantly in MSCs treated with *ad*Oct4 (*p* < 0.001, Fig. 7B, C). Moreover, these changes were seen the greatest in *ad*Oct4 transfected MSCs cocultured with *ad*FoxC1-treated ECs and *si*Oct4 reversed these changes. Our previous findings demonstrated that Oct4 could regulate the expression of its downstream genes, including Ang1, bFGF, and VEGF, which promotes angiogenesis [53]. Therefore, based on the results of the antibody array, we selected Ang1, bFGF, HGF, and VEGF for ELISA detection in MSCs induced for Oct4 overexpression or not to validate the antibody array results. We analyzed the concentration changes of these cytokines in MSCs before and after Oct4 treatment. The levels of these target factors declined gradually in MSCs treated with *si*Oct4. However, this decrease was completely inhibited in MSCs treated with *ad*Oct4, and the levels of these target factors were increased dramatically in MSCs cocultured with *ad*FoxC1-treated ECs after Oct4 overexpression (Fig. 7D, E, F).

Moreover, immunocytofluorescence staining confirmed the increased levels of these cytokines after Oct4 overexpression (Fig. 7G). This suggested that overexpression of Oct4 induced angiogenesis by upregulating its related downstream pro-angiogenic cytokines in MSCs under hypoxia.

### FoxC1 mediates MEndoT in the ischemic heart

**Fig. 8:**
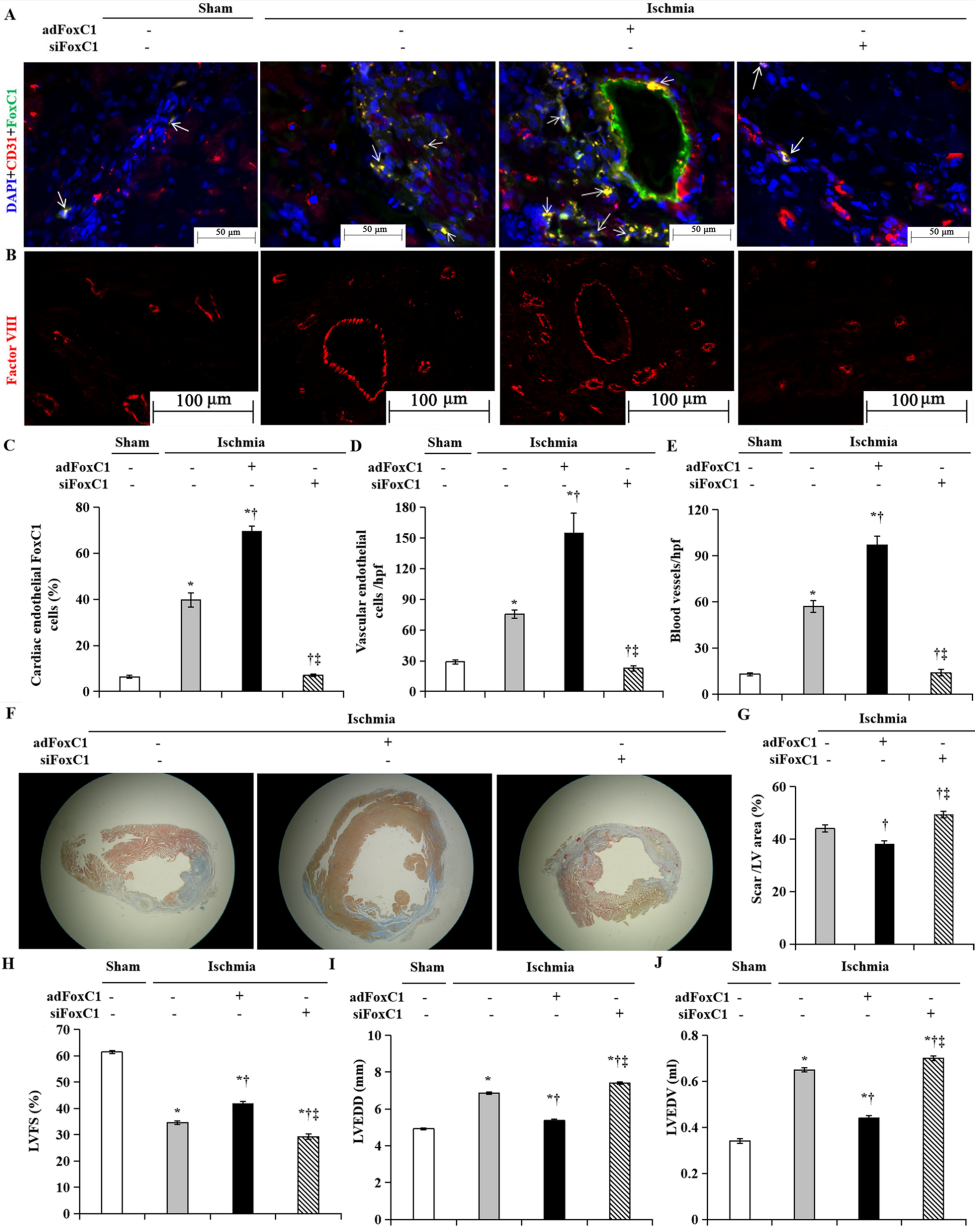
MEndoT after cardiac injury is FoxC1 dependent. **A** FoxC1 and CD31 immunostaining in the sham operation and the ischemic hearts in the presence or absence of *ad*FoxC1 transfection (**A**) and **C** quantification of FoxC1 expression in endothelial cells. The greatest amount of cardiac endothelial FoxC1^+^ cells was found in the FoxC1 overexpressed ischemic hearts (arrowheads). Scale bar: 50 μm. **B** Factor VIII immunostaining in these hearts. Scale bar: 100 μm. **D**, **E** Number of endothelial cells/high power field (**D**) and blood vessels (**E**). **F** Masson’s Trichrome staining 30 days after myocardial ischemia. **G** Quantification of scar/LV area. **H** Cardiac function and structure were assessed by left ventricular fraction shortening (LVFS), and LV remodeling indices including LVEDD (**I**) and LVEDV (**J**) 15 days after ischemia. All data are the means ± SEM. *p* < 0.05: *versus the heart receiving sham-ischemic operation, ^†^versus the ischemic hearts without FoxC1 intervention, ^‡^versus the ischemic hearts with *ad*FoxC1 transfection (*n* = 10 per group). Welch ANOVA analyses were performed in **C**, **D**, **E** and **I**, and one-way ANOVA analysis was used in** G**, **H** and **J**

To determine whether FoxC1 mediates MEndoT in vivo, we subjected rats to ischemic cardiac injury by ligation of the left anterior descending coronary artery. The hearts were transfected with vectors encoding FoxC1 (*ad*FoxC1(+)), FoxC1 *si*RNA (*si*FoxC1(+)), or control vectors (−). Rats which were not ligated in the same part of the hearts and received no *ad*FoxC1 or *si*FoxC1 were served as sham operation group. Compared with the sham group, ischemia resulted in a significant increase in cardiac vascular endothelial FoxC1 levels 15 days after cardiac injury, and the effect was the greatest in the *ad*FoxC1 group, but in rat with FoxC1 deletion, the FoxC1 expression in border zone cardiac endothelial cells failed to increase significantly (Fig. 8A, C). FoxC1 overexpression resulted in 4.3 fold and 1.1 fold increase of MEndoT compared with the sham-operated group and the *ad*FoxC1(+) group (Fig. 8A, D), and was associated with an increase in capillary density in the injury region (Fig. 8B, E). Increase in vessel density in the injured heart is associated with cardiac repair after MI [34, 42]. Masson Trichrome staining demonstrated the greatest decrease of collagen deposition in the hearts of adFoxC1(+) animals (Fig. 8F, G). Echocardiography on hearts of mice 15 days after cardiac injury showed significant improvement of cardiac function (LVFS, Fig. 8H) and amelioration of the LV end diastolic diameter (LVEDD, Fig. 8I) and the LV end diastolic volume (LVEDV, Fig. 8J) in the adFoxC1(+) animals. Conversely, in comparison with the sham and ischemic control animals, the depletion of FoxC1caused the decrease of LVFS and the dilation of both LVEDD and LVEDV. These observations demonstrate that FoxC1 is necessary for MEndoT to occur after cardiac ischemia and that disruption of MEndoT is associated with the decline of angiogenesis and cardiac function and the deterioration of cardiac remodeling.

### Activation of FoxC1/Oct4 axis enhances MEndoT of MSCs in ischemic hearts

**Fig. 9:**
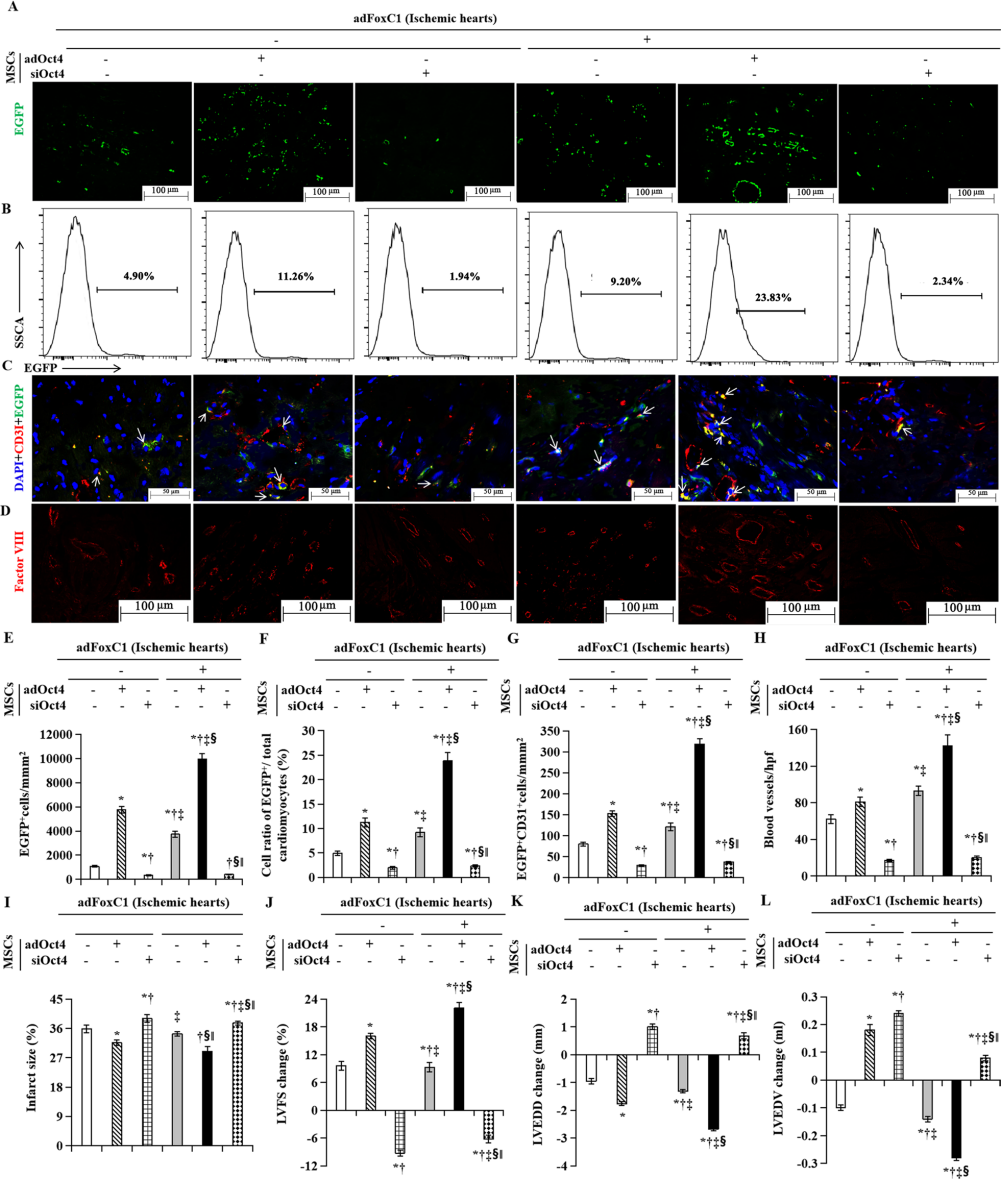
Oct4 enhances FoxC1-mediated MEndoT after MSC therapy. **A** Representative fluorescence microscopy images of tissue sections showing the retention of EGFP labeling MSCs at the injection site 30 days after transplantation. Scale bar: 100 μm. **E** Quantitative data showing the retention of EGFP^+^ MSCs in the ischemic hearts. **B** Representative phenotype of gated EGFP^+^ cells evaluated by FACS. **F** Quantitative analysis of the percentages of EGFP-positive cells (EGFP^+^) relative to the whole ventricular cell population in the ischemic hearts after 30 days of transplantation. **C**, **G** Endothelial cell immunostaining in EGFP-labeled MSCs (**C**, arrowheads) and quantitation of endothelial cell expression (**G**). Scale bar: 50 μm. **D**, **H** Vascular density images assessed by factor VIII immunostaining (**D**) and statistical analysis of blood vessels (**H**) in various groups 30 days after cell therapy, respectively. Scale bar: 100 μm. (**I**) Quantitation of infarct size. **J**, **K**, **L** The changes of LVFS (**J**), LVEDD (**K**), and LVEDV (**L**) prior to and 30 days after cell therapy. All graphs show means ± SEM. *p* < 0.05: in the ischemic hearts without FoxC1 transfection, *versus MSC therapy alone, ^†^versus Transplantation of *ad*Oct4 transfected MSCs, ^‡^versus Transplantation of *si*Oct4 transfected MSCs; in the *ad*FoxC1 hearts, ^§^versus MSC therapy alone, ^||^versus Transplantation of *ad*Oct4 transfected MSCs (*n* = 10 per group). Welch ANOVA analyses were performed in **E**–**I** and **K**, and one-way ANOVA analysis was used in **J** and** L**

We next rescued MEndoT in cardiac MSC-engrafted ischemic microenvironments. Ad.FoxC1 animals or control vectors (−) animals were injected with MSCs transfected with *ad*Oct4, *si*Oct4, or control vectors at 15 days post-MI, and followed up for 30 days. First, we investigated the relationship between FoxC1/Oct4 axis and transplanted MSC survival/engraftment. The detection of EGFP-labeled MSCs was confirmed by immunofluorescence and FACS. Cell retention 30 days after the transplantation of MSCs into these ischemic hearts is shown in Fig. 9A. EGFP^+^ cells were found more in the *ad*FoxC1 ischemic hearts than in the control vectors (−) ischemic hearts. MSCs with *ad*Oct4 exhibited much higher engraftment in the *ad*FoxC1 hearts compared with the control hearts (Fig. 9A, E). Consistently, FACS demonstrated that depletion of Oct4 reduced significantly the retention of survival MSCs in both the adFoxC1 ischemic hearts and the control vectors (−) ischemic hearts. However, Oct4 transfection significantly increased the retention of EGFP^+^ MSCs, especially in the *ad*FoxC1 ischemic hearts (Fig. 9B, F), indicating an increase in cell engraftment in the overexpression of FoxC1/Oct4.

We next explored whether stimulation of FoxC1/Oct4 axis after cardiac ischemia enhances MEndoT of MSCs. We found that labeled MSCs in the *ad*FoxC1 ischemic hearts exhibited significantly higher vascular cell expression than in the control hearts, and overexpression of Oct4 further enhanced the degree of this MEndoT. Conversely, in Oct4-depleted MSCs, the MEndoT was dramatically reduced (Fig. 9C, G). Similar results were obtained in neovascularization of MSCs: As compared with control, FoxC1-transfected animals have significantly higher blood vascular density in the ischemic hearts, and Oct4 overexpression could promote blood vessels, whereas depletion of Oct4 abolished this effect (Fig. 9D, H), suggesting that FoxC1-mediated MEndoT promotes cardiac MSC neovascularization via Oct4 signaling.

Moreover, overexpression of FoxC1/Oct4 axis was also associated with decrease of inflammatory and fibrosis measured by HE staining, immunofluorescence, and masson trichrome staining. H&E staining showed that, in the ischemic hearts without adFoxC1 transfection, compared without Oct4 expression, Oct4 overexpressed MSCs therapy did significantly reduce MI-induced significant inflammation, including neutrophil infifiltration, myocyte loss, and bleeding. Transplantation of Oct4 overexpressed MSCs into the *ad*FoxC1 ischemic hearts caused the greatest reduction of myocardial inflammation. However, this reduction was abolished in the animals receiving *si*Oct4 MSCs (Additional file 5: Fig. S5A). The quantitative indices for inflammation, i.e., myocardium MPO and ROS, were highest in the control hearts receiving *si*FoxC1 transfected MSCs injection and were the lowest in the *a*dFoxC1 hearts receiving Oct4 transfected MSC therapy (Additional file 5: Fig. S5D, E). The degree of MSC transformation into inflammatory cells measured by the number of CD68 expressing macrophages was significantly smaller in the *ad*FoxC1 transfected ischemic hearts than in the control vectors (−) hearts, and Oct4 overexpression further reduced this transformation (Additional file 5: Fig. S5B, F). Masson Trichrome staining demonstrated the smallest amount of collagen deposition (Additional file 5: Fig. S5C) and infarct size (Fig. 9I) in the FoxC1 transfected hearts receiving *ad*Oct4-treated MSCs. Moreover, more salvaged myocardial tissue was observed in the *ad*FoxC1 ischemic hearts, and Oct4 overexpression further increased viable myocardiocytes. However, *si*Oct4 cancelled this decrease (Additional file 5: Fig. S5G). These observations suggest that FoxC1-mediated MEndoT contributes to preventing myocardial cell loss by inhibiting fibrosis and inflammation thorough upregulation of Oct4.

Of note, increased neovascularization, and decreased inflammatory and collagen deposition led to reduction in infarct size and improvement in post-injury cardiac function and remodeling. Echocardiography demonstrated that injection of Oct4 transfected MSCs significantly improved cardiac function and decreased LVEDD and LVEDV compared with MSC injection alone; the maximal improvement was observed in the *ad*FoxC1 transfected rats treated by Oct4 overexpressed MSCs; depletion ofOct4 abrogated this effect (Fig. 9J, K, L).

We next investigated the expression of FoxC1/Oct4 signaling in ischemic hearts. ELISA showed that FoxC1 and Oct4 expression increased in the FoxC1 transfected hearts, peaked in these hearts receiving transplantation of Oct4 overexpressed MSCs. However, Oct4 knockdown weakened this increase (Additional file 6: Fig. S6A, B). Overexpression or knockdown of Oct4 caused no significant change in FoxC1 expression in all the hearts without *ad*FoxC1 transfection, indicating that Oct4 is a downstream transcript factor of FoxC1 action.

The expressions of vascular growth factors including Ang-1, bFGF, and VEGF in the ischemic hearts were parallel to Oct4 expression, and their expression patterns were seen with the greatest significant increase in the *ad*FoxC1 ischemic hearts receiving Oct4 transfected MSCs (Additional file 6: Fig. S6C–E). Contrary to increased expression of growth factors, the expression of inflammatory factors IL-6 and TGFβ1 was substantially reduced in Oct4 transfected MSCs injected animals, and showed the lowest expression in the *ad*FoxC1 ischemic hearts receiving Oct4 transfected MSCs, while depletion of Oct4 canceled this reduction (Additional file 6: Fig. S6F, G). Conversely, we found transplantation of Oct4 transfected MSCs caused the greatest release of anti-inflammatory factor IL-4 in the *ad*FoxC1 rats, the secondary in the control rats, transfection of *si*Oct4 into MSCs markedly decreased IL-4 expression (Additional file 6: Fig. S6H). These observations suggest that FoxC1/Oct4 axis contributes to inhibiting cardiac inflammation of MSCs in the ischemic hearts. Taken together, the FoxC1/Oct4 axis in this model simultaneously initiates an anti-inflammation by directly affecting expression of inflammatory specific factors.

## Discussion

Delivery of MSCs in the absence of a cytoprotective environment offers limited efficacy due to low cell retention and poor graft survival [41]. Recent studies have shown that FoxC1 promote cell proliferation and self-renewal of stem cells in ischemic brain [26] Several reports have confirmed a protective function of vascular niches in adult stem cells [14] and a creation of nurturing niches for stem cells [7]. In this context, the present in vitro study shows that FoxC1 induces hypoxic ECs to possess a degree of native vascularization, e.g., mimicking the ischemic niche, which enables MSCs to adopt endothelial cell like fates after hypoxia, and beget MEndoT. Overexpression of Oct4 further augmented the proliferation and vascular formation of MSCs, accomplishing through activation of angiogenesis pathways. Specific Oct4 deletion in MSCs reduced their survival and MEndoT in the hypoxic ECs with FoxC1 transfection. Overexpression of FoxC1 in the ischemic hearts induced a vascular niche, and further augmented survival and MEndoT of MSCs after administration of *ad*Oct4 transfection. Enhanced MEndoT induced by the FoxC1/Oct4 axis appears to play an important physiological role in cardiac repair, as disruption of MEndoT by depletion of Oct4 worsened post-infarct vascularity and cardiac function.

It has been shown that preexisting endothelial cells mediate cardiac neovascularization after MI [18]. We show here that MI in rat models resulted in characteristic histological and humoral changes consisting of angiogenesis and pro-angiogenic factor expression. These changes are believed to represent an intrinsic adaptive formation of a vascular niche accelerating stem cell survival and function [52]. However, these response showed short-term time. Thus, elucidation of signaling pathways that modulate vascular formation of vascular ECs is important for the maintenance and long-term survival of MSCs engrafted into the MI. We showed here that hypoxic ECs expressed FoxC1, and followed by a high expression of pro-angiogenic and transcription factors involved in stem cell self-renewal. In contrast, these factors were expressed at very low levels in ECs cultured under normoxia. Consistent with the in vitro observation, a similar expression pattern was evidenced in the infarcted hearts: FoxC1 was highly expressed in the peri-infarct (ischemic) area than in the remote myocardium (normoxic area). Specific FoxC1 deletion in hypoxic ECs resulted in defective clone-intitiating potential. FoxC1 is required for haematopoietic stem/progenitor cell niche formation [37]. These data indicate that FoxC1 is an essential factor in early expansion of preexisting vessels in a damaged heart, namely development of blood vascular niche.

The transcription factor FoxC1 can favor a vascular fate by inducing differentiation of somite-derived ECs in the limb [35]. Here, we show that FoxC1 is essential for controlling ECs proliferation and differentiation, as well as the expression of numerous angiogenesis-related factors in the hypoxic microenvironment. FoxC1 deletion led to obvious reduction of vascular formation. Once again demonstrating that the contribution of preexisting ECs to vascularization is dependent on FoxC1 expression. In addition, we observed smaller changes in proliferation and vascular formation of ECs under normoxic condition after treatment with *ad*FoxC1/*si*FoxC1. Therefore, we provided novel insights into FoxC1 activation in the preexisting ECs, which might represent another signaling pathway to maintain the vascular microenvironment under hypoxia.

However, the molecular mechanisms within the vascular microenvironment remain largely unknown. In the present study, in vitro analysis showed that preexisting ECs promote MSC self-renewal under hypoxic conditions. FoxC1 levels in ECs were inversely proportional to the MSC apoptosis rate, and diminished expression of FoxC1 in knockdown state significantly increased MSC apoptosis. FoxC1-transfected ECs significantly promoted MSC proliferation and self-renewal in comparison with ECs coculture alone, indicating that FoxC1 regulates MSC survival and growth. The importance of FoxC1 in stem cell survival and growth was consistent with the results reported by Lee et al., who identified that activated FoxC1 promoted cell proliferation and self-renewal of multipotent arachnoid-pia stem cells [26]. We also found that overexpression of FoxC1 in ECs was linked with increased expression of cytoprotective molecules, including pro-angiogenic cytokines Ang1, bFGF, and VEGF, and anti-apoptotic factor Bcl2, and decreased expression of apoptotic proteins Bax and Caspase3 involved in survival and proliferation of MSCs [53]. Taken together, these findings implied that vascular microenvironment with higher levels of FoxC1 expression are essential to maintain MSC survival and self-renewal under hypoxia.

Although FoxC1 has recently been identified as an efficient approach to preserve long-term survival of hair follicle stem cells [25], in this study, we found that the proliferation is not the case in the hypoxia-cultured MSCs. The coculture of MSCs with *ad*FoxC1-treated ECs could prolong the MSC doubling time compared with ECs alone, whereas the number of population doublings of cultured MSCs gradually decreased after 84 h of hypoxic culture. Therefore, modulation of FoxC1 signaling pathways in ECs appears to be crucial to develop strategies to induce long-term survival of MSCs against hypoxic or ischemic injury. Several signaling pathways, such as PI3K/AKT, MAPK/ERK [30], HIF-1, VEGF [38], and JAK2/STAT3 [45] pathways, are implicated in regulating MSC survival, self-renewal, and angiogenesis. It has been found that FoxC1 knockdown reduced stem cell percentage, suppressed self-renewal ability, decreased expression of stemness-related genes (Oct4, NANOG, SOX2 and ABCG2) [5]. Among them, we identified that following activation of FoxC1 in ECs, only Oct4 is produced at high levels in MSCs. However, other stem cell factors showed no significant change in response to alteration of FoxC1 expression, suggesting that FoxC1 can specially and significantly activate Oct4 expression. Moreover, overexpression of Oct4 further enhanced self-renewal of the MSCs cocultured with *ad*FoxC1-treated ECs, similar to the long-term survival of role for Oct4 described previously in ESCs [29]. All these data indicated that Oct4 plays a critical role in the regulation of MSC growth and survival.

It has been found that knockout of Oct4 in perivascular cells decreases angiogenesis following hindlimb ischemia [19]. Here, we observed that overexpression of Oct4 stimulated MSC angiogenesis, namely MEndoT, which in turn enhanced MSCs’ repopulating ability and proliferation, and reduced apoptosis. MEndoT, which has been confirmed to play an important physiological role in cardiac repair, provides an efficient strategy to rapidly increase neovascularization in ischemic hearts [42]. Several publications have confirmed or corroborated the existence of MEndoT, or provided additional observations of its effects. The main origin of MEndoT-derived cells has been identified as cardiac fibroblasts [13]. In the present study, Oct4 improved the acquisition of an endothelial-like phenotype of MSCs in the FoxC1-induced vascular microenvironment. Upregulation of Oct4 in MSCs improved MEndoT and increased neovascularization after hypoxia. In particular, the release of FoxC1 in the vicinity of blood vascular ECs might result in a more hospitable niche, and facilitate MSC angiogenesis, associated with inhibition of fibrosis. Moreover, the FoxC1-driven transcriptional program that drives MEndoT under hypoxia is impaired in Oct4-deficient MSCs, and a high fibrosis rate is a prominent feature of *si*Oct4-MSCs. This result indicates that the effects of FoxC1 on MEndoT of MSCs was Oct4 dependent. This phenomena of MSC MEndoT was disproved by Batlle’s study, which indicated that p38α in MSCs regulates a TGF-β-induced angiogenesis program negatively, including their ability to transdifferentiate into endothelial cells [3]. All these data suggest that Oct4-mediated MEndoT might represent a therapeutic target to improve MSC fate under hypoxic conditions.

Oct4 acts as a gatekeeper in increasing the proliferation of germline stem cells under hypoxic stress [46], and has a critical protective role in perivascular cell migration and recruitment during injury- and hypoxia-induced angiogenesis [19]. However, how Oct4 regulates MSCs remains largely unknown. Previous studies have shown that increased expression of Oct4 in bone marrow MSCs under hypoxic or ischemic conditions improved cell survival via increased expression of a variety of hypoxia-responsive factors, such as Ang1, bFGF, and VEGF [47, 53]. Here, we found that Oct4 deletion in MSCs abolished increased expression of a set of pro-angiogenic cytokines, Ang1, bFGF, and VEGF, and anti-inflammatory factors IL4 and IL10 induced by coculture with *ad*FoxC1-treated ECs. These growth factors are key in survival and angiogenesis of MSCs [53, 55]. But, in contrast, Oct4 deletion resulted in increased expression of pro-inflammatory (IL6) and pro-fibrotic (TGFβ1) cytokines critically involved in fibrosis [12]. This change is correlated with reduced MEndoT and increased fibrosis in MSCs under hypoxia. These data indicated that FoxC1 drives survival and vascularization of hypoxic MSCs downstream of Oct4. Human Oct4 can be alternatively spliced and generate Oct4A, Oct4B, and Oct4B1 [32]. Oct4A protein is a transcription factor for the stemness of embryonic stem cells (ESCs), while the function of Oct4B isoforms remains unclear [28]. In the present study, the anti-Oct4 antibody was used to detect the overall expression of Oct4, which cannot distinguish their respective expression between these three free monomers. Which isoform of Oct4 is associated with increased vascularization of MSCs remains for further investigation.

Two mechanisms for FoxC1/Oct4 axis on determining the fate of MSCs in ischemic hearts are conceivable. On the one hand, a high degree of FoxC1 expression produced a high concentration of pro-angiogenic cytokines and stimulates expansion of preexisting ECs, which contributed to the formation of blood vessels, indicating contribution of MEndoT to neovascularization [46]. In line with this, overexpression of FoxC1 induced inhibition of cardiac remodeling and resulted in improvement of cardiac function. On the other hand, sustained activation of FoxC1 provided a vascular niche which benefited the survival and function of MSCs transplanted into ischemic hearts [54]. Our results in ischemic hearts supported the hypothesis of an anti-apoptosis-inducing effect of FoxC1 at a high expression rate, while a lower expression rate decreased engraftment of MSCs. A dual role of Oct4 has also been demonstrated in the ischemic hindlimb muscle [19]. Here Oct4 promoted the survival of MSCs and FoxC1-induced MEndoT at high expression levels in the ischemic hearts. In the FoxC1-induced vascular niche, Oct4 was upregulated in ischemic cardiomyocytes to an optimal extent to activate cytoprotective factors such as Ang1, bFGF, VEGF, and IL4. This led to increased cardiac function and inhibited cardiac remodeling. In contrast, Oct4 deficiency mediated activation of the IL6/TGFβ1 pathway with pro-inflammatory and fibrotic effects by increasing inflammatory and collagen transformation [43]. This may unveil a notice mechanism: These cytoprotective factors may constitute an exosome which provides cytoprotection and modulates apoptosis [8]: FoxC1-mediated exosome may activate an angiogenesis program in improving MEndoT after hypoxia/ischemia by activating the Oct4 signaling pathway. This will be an important subject for future investigation. Our findings provide further rationale for the ongoing clinical evaluation of combinatorial therapies comprising genetic modification and MSC transplantation [10]. Although several investigations in the present study were done extensively with rigor to explore how FoxC1/Oct4 axis impacts MSCs under ischemic conditions, the translational implication and the therapeutic potential of FoxC1 on MSCs post-infarction are not yet clear. Further experiments need to investigate the impact of overexpression of FoxC1 within MSCs themselves, and how such genetic modification of MSCs could enhance their therapeutic potential for myocardial infarction.

## Conclusions

In summary, we show by a vascular niche model that FoxC1 is an essential regulator of MSC responses in the hypoxic microenvironment. Furthermore, we show that this occurs FoxC1 regulation of Oct4; and that Oct4 in the ischemic hearts overexpressing FoxC1 contributes to MSC survival, vascularization, and cardiac repair. Investigation of such cytoprotective mechanisms should provide potential therapeutic targets for the future treatment of ischemic diseases.

## Supplementary Information

**Additional file 1: Fig. S1.** The treatments flowchart and the cell and animal groups.

**Additional file 2: Fig. S2.** Surface marker expression of rat MSCs.

**Additional file 3: Fig. S3.** Serial changes of blood vascular density and pro-angiogenic cytokines in the ischemic areas after MI induction.

**Additional file 4: Fig. S4.** FoxC1 deficiency impairs self-renewal of ECs under hypoxia.

**Additional file 5: Fig. S5.** Exhibits Oct4 enhancing MSC-mediated amelioration of MI pathology.

**Additional file 6: Fig. S6.** ELISA assay of difference in FoxC1, Oct4, Ang1, bFGF, VEGF, IL-6, TGF-β1, and IL-4 at day 30 post-MI in various groups.

**Additional file 7: Table S1.** The qRT-PCR primers.

**Additional file 8: Table S2.** The antibodies for western blotting, immunofluorescence, and immunohistochemistry, respectively.

**Additional file 9.** An expanded Materials and Methods section.

## Data Availability

The datasets used and/or analyzed during the current study are available from the corresponding author on reasonable request.

## Abbreviations

acLDL: Acetylated low-density lipoprotein
α-SMA: Alpha-smooth muscle actin
Ang1: Angiopoietin1
Bax: BCL2-associated X
BCLF1: Bcl-2-associated transcription factor 1
bFGF: Basic fibroblast growth factor
BrdU: 5-Bromodeoxyuridine
CD: Cluster of differentiation
DAPI: 4′,6-Diamidino-2- phenylindole
ECs: Endothelial cells
EF: Ejection fraction
ELISA: Enzyme-linked immunosorbent assays
ESCs: Embryonic stem cells
FACS: Fluorescence-activated cell sorting
FOX: Forkhead/winged-helix transcription factor
FoxC1: Forkhead box C1
FS: Fractional shortening
GAPDH: Glyceraldehyde-3-phosphate dehydrogenase
H&E: Hematoxylin and eosin
HGF: Hepatocyte growth factor
HIF-1α: Hypoxia-inducible factor 1 alpha
HO: Hypoxia
IHC: Immunohistochemistry
Klf4: Kruppel-like factor 4
LV: Left ventricular
LVEDD: LV end diastolic diameter
LVEDV: The LV end diastolic volume
MACS: Magnetic-activated cell sorting
MEndoT: Mesenchymal-to-endothelial
MI: Myocardial infarct
MSCs: Mesenchymal stem cells
NO: Normoxic condition
Oct4: Octamer-binding protein4
qRT-PCR: Quantitative real-time reverse transcription PCR
SH2: Src homology domain 2
SH3: Src homology domain 3
shRNA: Short hairpin RNA
SP1: Specificity protein 1
TFs: Transcription factors
TGFβ1: Transforming growth factor beta1
TTC: Triphenyltetrazolium chloride
TUNEL: Terminal deoxynulceotidyl transferase nick-end labeling
UEA-1: Ulex europaeus agglutinin-1
VEGF: Vascular endothelial growth factor
VEGFR2: Vascular endothelial growth factor receptor 2
vWF: Von Willebrand factor
