# Supplementary figures and images for: Oct4-dependent FoxC1 activation improves the survival and neovascularization of mesenchymal stem cells under myocardial ischemia
Source: Stem Cell Res Ther. 2021 Aug 28;12:483. doi: 10.1186/s13287-021-02553-w (PMC8403428; doi:10.1186/s13287-021-02553-w)

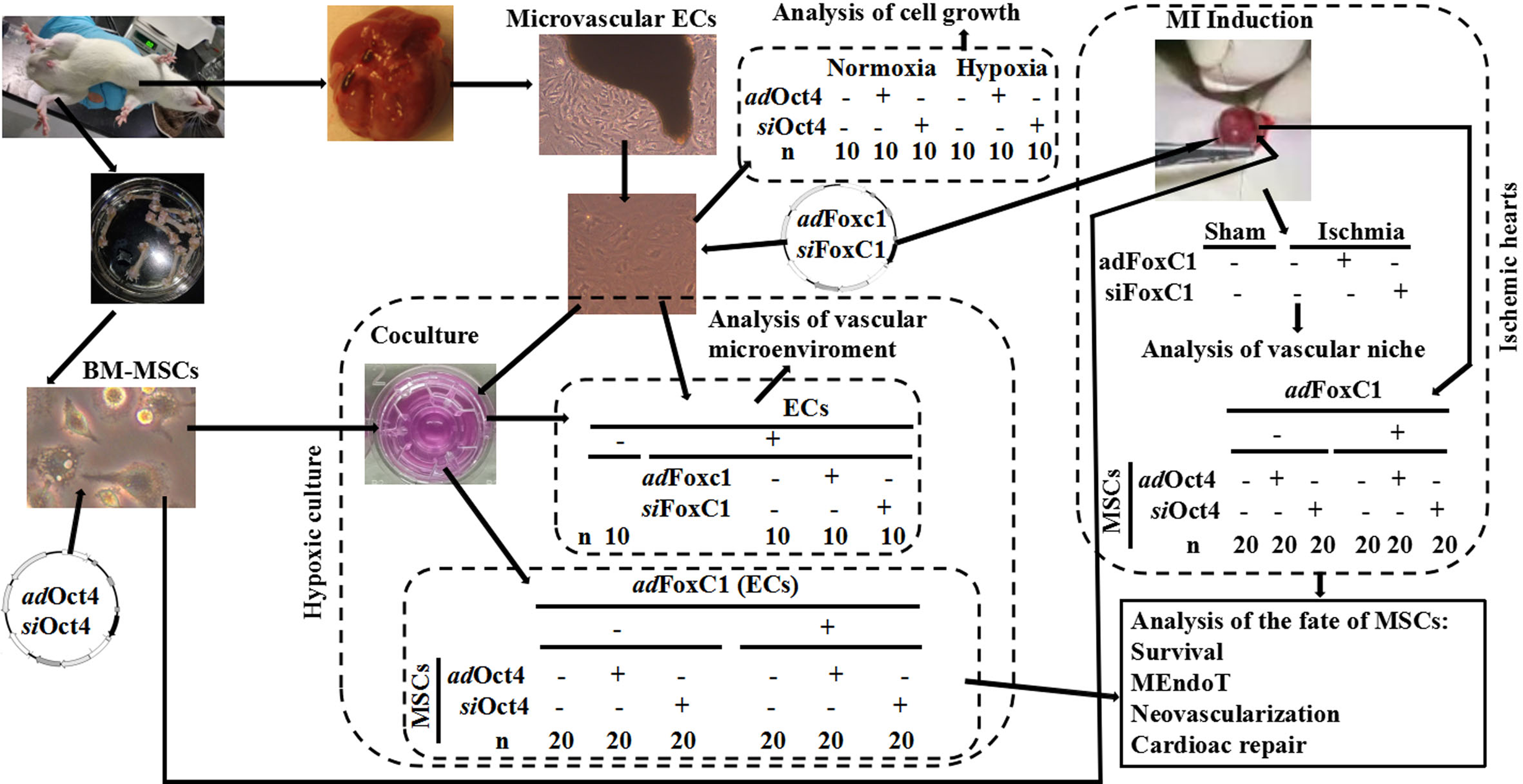

Supplement: Supplementary file 1 — Additional file 1: Fig. S1. The treatments flowchart and the cell and animal groups. [file 13287_2021_2553_MOESM1_ESM.tif]

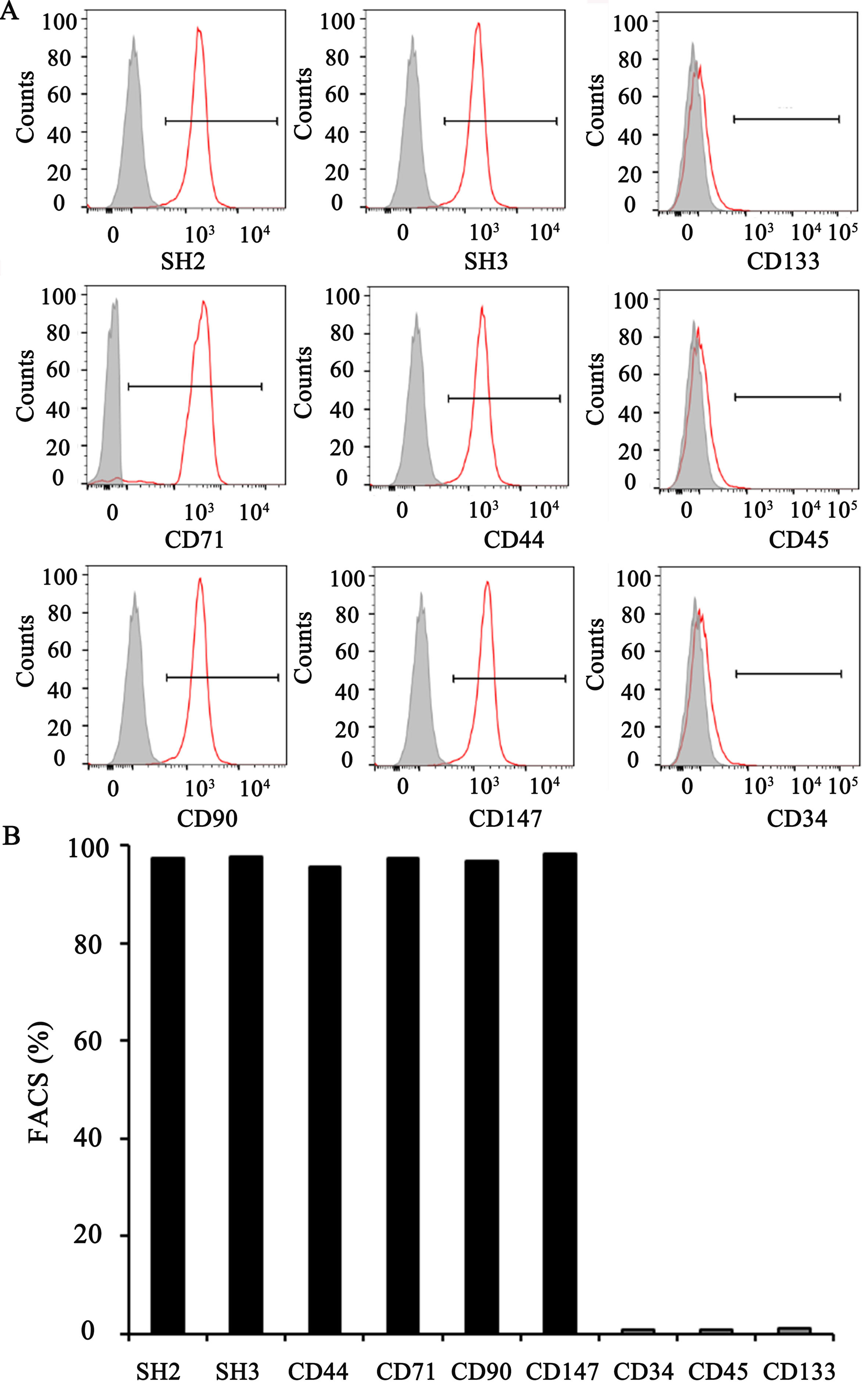

Supplement: Supplementary file 2 — Additional file 2: Fig. S2. Surface marker expression of rat MSCs. [file 13287_2021_2553_MOESM2_ESM.tif]

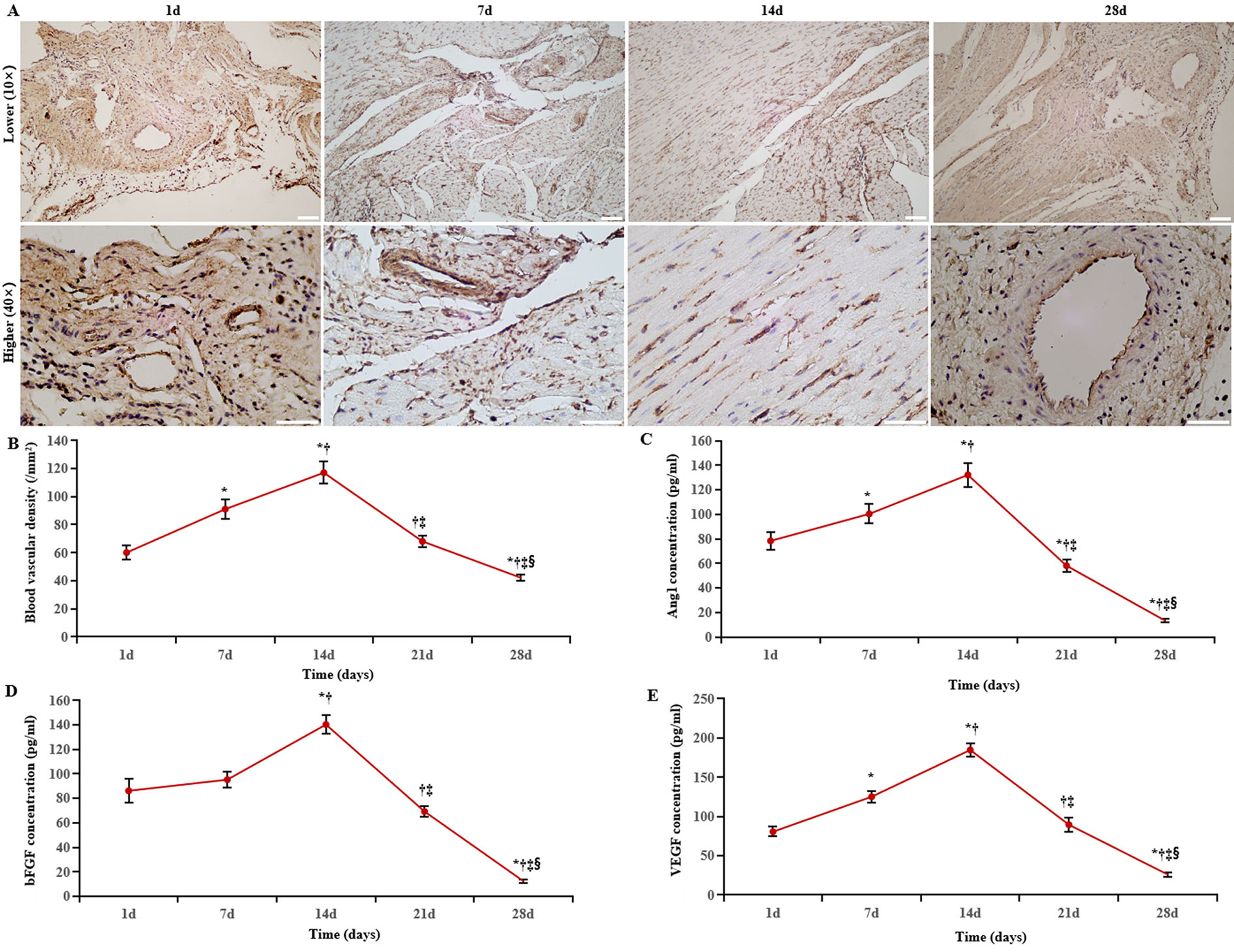

Supplement: Supplementary file 3 — Additional file 3: Fig. S3. Serial changes of blood vascular density and pro-angiogenic cytokines in the ischemic areas after MI induction. [file 13287_2021_2553_MOESM3_ESM.tif]

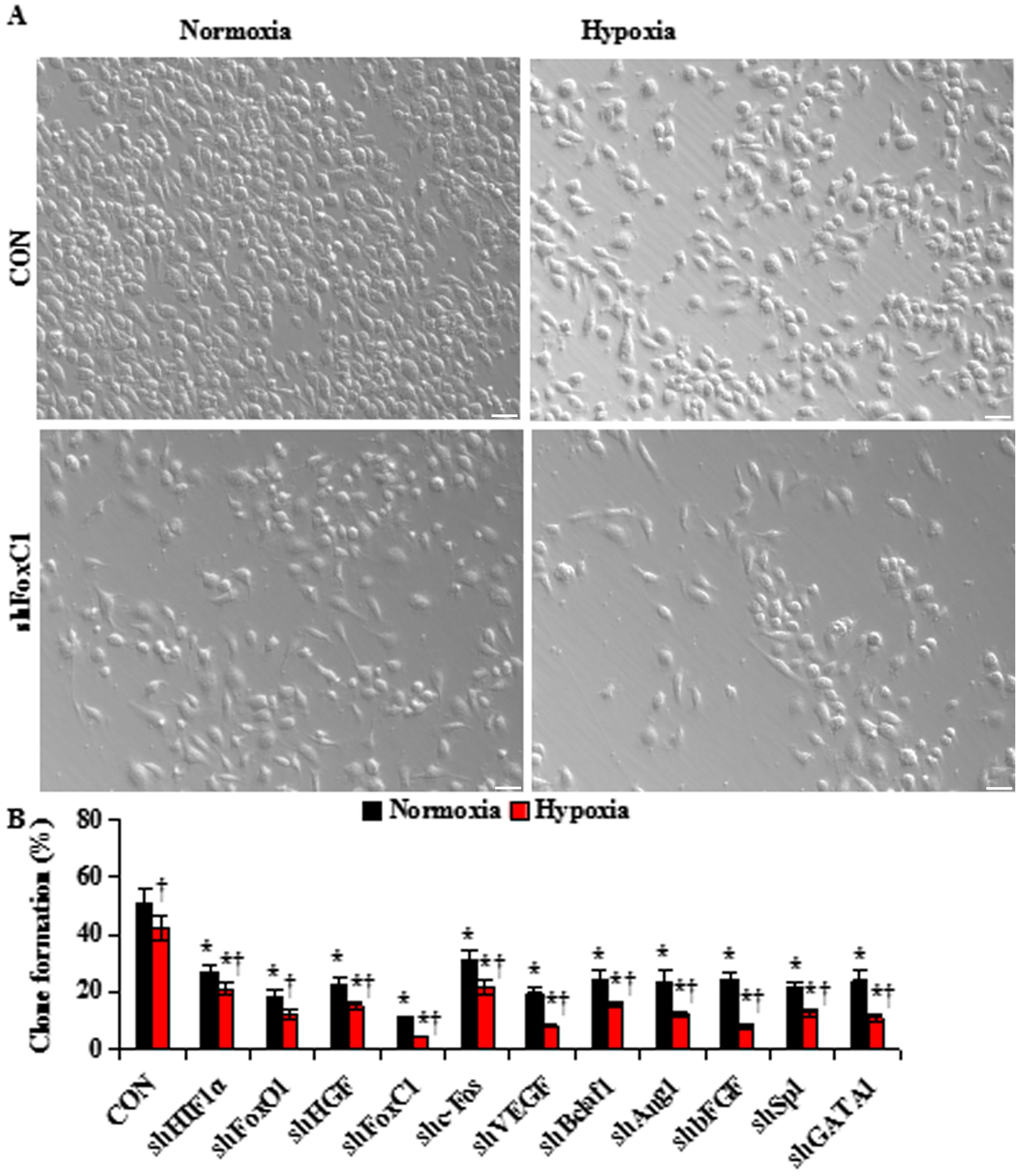

Supplement: Supplementary file 4 — Additional file 4: Fig. S4. FoxC1 deficiency impairs self-renewal of ECs under hypoxia. [file 13287_2021_2553_MOESM4_ESM.tif]

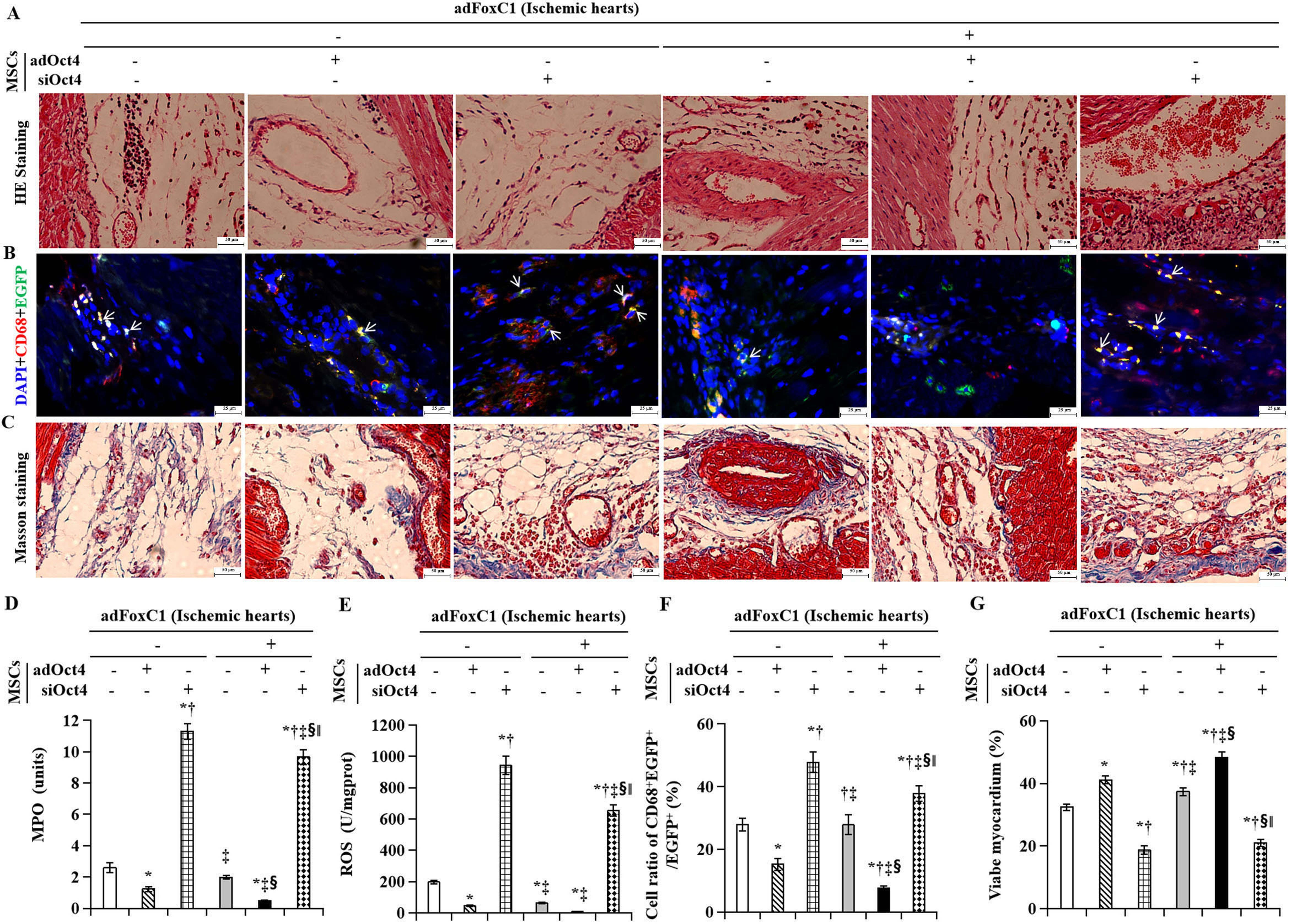

Supplement: Supplementary file 5 — Additional file 5: Fig. S5. Exhibits Oct4 enhancing MSC-mediated amelioration of MI pathology. [file 13287_2021_2553_MOESM5_ESM.tif]

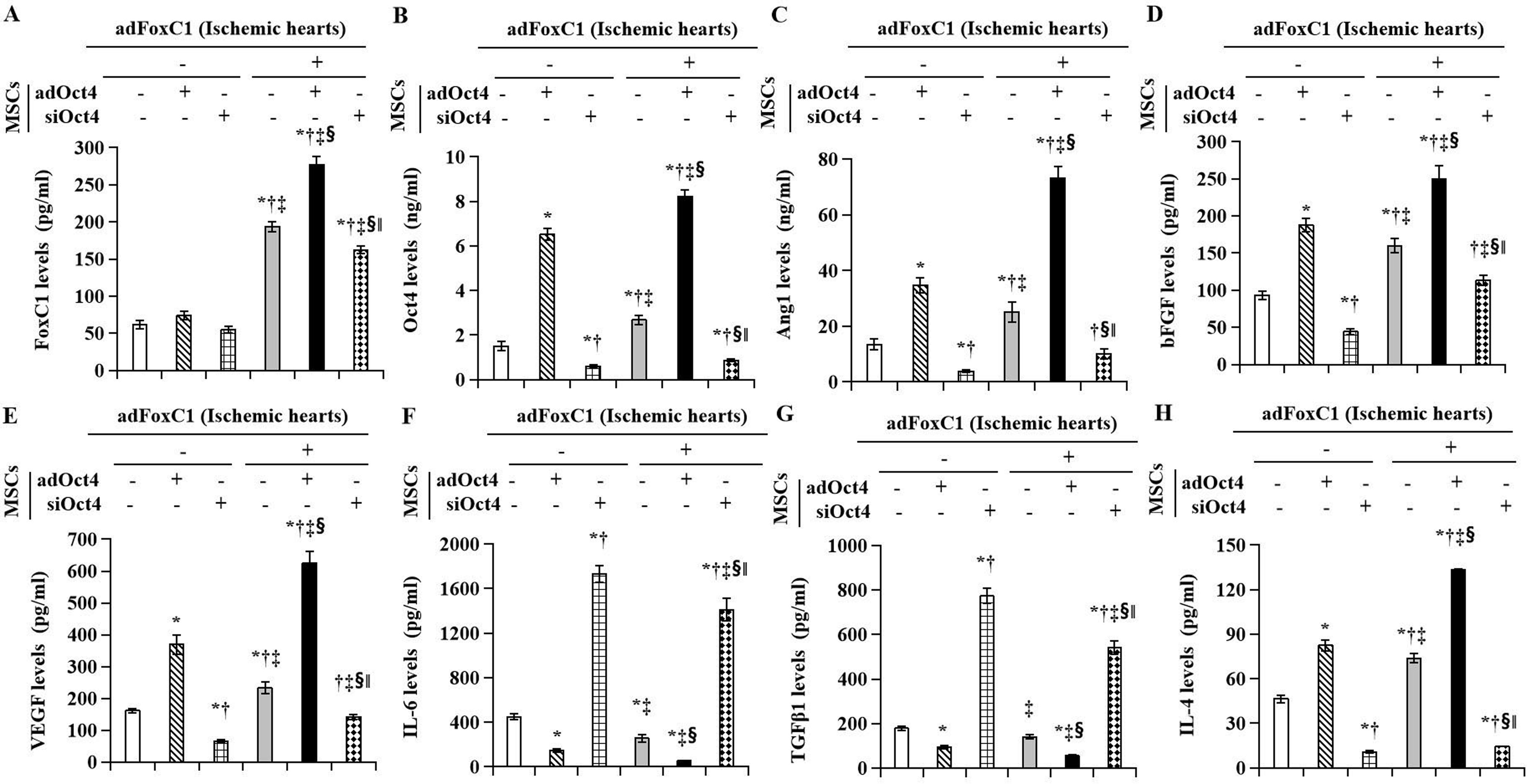

Supplement: Supplementary file 6 — Additional file 6: Fig. S6. ELISA assay of difference in FoxC1, Oct4, Ang1, bFGF, VEGF, IL-6, TGF-β1, and IL-4 at day 30 post-MI in various groups. [file 13287_2021_2553_MOESM6_ESM.tif]
