# Supplementary material for: Oct4-dependent FoxC1 activation improves the survival and neovascularization of mesenchymal stem cells under myocardial ischemia
Source: Stem Cell Res Ther. 2021 Aug 28;12:483. doi: 10.1186/s13287-021-02553-w (PMC8403428; doi:10.1186/s13287-021-02553-w)
Supplement: Supplementary file 7 — Additional file 7: Table S1. The qRT-PCR primers. [file 13287_2021_2553_MOESM7_ESM.docx]

**Table S 1 The primers for real-time RT-PCR**

| Gene | Description | Primer Sequence | Product  size (bp) |
| --- | --- | --- | --- |
| Foxc1 | Forkhead box C1 | AAGACGGAGAACGGTACGTG  CGGGACTCTCGATTTTAGGCA | 103 |
| FoxO1 | Forkhead box O1 | CCCAGGCCGGAGTTTAACC  GTTGCTCATAAAGTCGGTGCT | 132 |
| HIF-1α | Hypoxia inducibel factor 1 alpha | TCATCAGTTGCCACTTCCCC  AGCAGTTCATCTGTGCTTTCA | 149 |
| Oct4 | Octamer-binding protein 4 | CCACTTCACCACACTCTACT  GTCACCGCATGTTAGAAGAC | 127 |
| c-Fos |  | CAGCTCCCACCAGTGTCTAC  TCGGCTGGGGAATGGTAGTA | 162 |
| Ang-1 | Angiopoietin 1 | AACAGGAGGTTGGTGGTTTGATG  GAACATCCCCAGATTTATTTCAGGT | 217 |
| bFGF | basic fibroblast growth factor | CCATCAAGGGAGTGTGTGCG  CCCAGTTCGTTTCAGTGCCA | 176 |
| VEGF | Vascular endothelial growth factor | CCGACAGGGAAGACAATGGGA  GGGATGGGTTTGTCGTGTTTCT | 149 |
| HGF | Hepatocyte growth factor | AAACTTCTGCCGGTCCTGTT  TGGTAAGAGTAGTTTTTGCTGACT | 142 |
| BCLF1 | Bcl-2-associated transcription factor 1 | TCGGCGTGTCAGGAATTTGA  ACTGTGATCTAGACTTGGATCTTGA | 181 |
| SP1 | Specificity Protein 1 | TTGGTGGCAATAACGGGGG  CTCGGACCCTGGGAGTTGTT | 199 |
| GATA1 | Globin transcription factor 1 | GATTCTGCCCTGGTGTCCTC  CGTGGCTGCATTTGGAGAAG | 105 |
| c-Myc |  | GGGATCCGGAGTCGCAGTAT  CTGGAGGCAAAGCCCTTCTC | 178 |
| Nanog |  | GTCCCTTCCCTTGCCGTTG  AGGAATCCCCAGAGTTCGATG | 96 |
| Klf4 | Kruppel-like factor 4 | GGGAAGGGAGAAGACACTGC  CCACTTTCCAGGTCTGTGGC | 141 |
| Vimentin |  | CAGCTCCAACCGGAGCTATG  GACCGGGTCACATAGGCG | 123 |
| Collagen I |  | CATGTTCAGCTTTGTGGACCTC  CCGTGCCATTGTGGCAGATA | 176 |
| MMP2 | matrix metalloproteinase2 | TTGACGGTAAGGACGGACTC  GGCGTTCCCATACTTCACAC | 134 |
| MMP9 | matrix metalloproteinase9 | AAGGGCGTCGTGGTTCCAACTC  AGCATTGCCGTCCTGGGTGTAG | 210 |
| GAPDH | Glyceraldehyde-3-phos-phate dehydrogenase | CCGAGGGCCCACTAAAGG  TGCTGTTGAAGTCACAGGAGACA | 67 |
