# Supplementary material for: Oct4-dependent FoxC1 activation improves the survival and neovascularization of mesenchymal stem cells under myocardial ischemia
Source: Stem Cell Res Ther. 2021 Aug 28;12:483. doi: 10.1186/s13287-021-02553-w (PMC8403428; doi:10.1186/s13287-021-02553-w)
Supplement: Supplementary file 9 — Additional file 9. An expanded Materials and Methods section. [file 13287_2021_2553_MOESM9_ESM.doc]

**Research design and methods**

**Antibodies and other reagents**

Transwell six-well plates were obtained from COSTAR The ABsolute QPCR SYBR Green premix was purchased from Takara. Supplementary **Tab. S1** presents the sequences of primers used in this study. Supplementary **Tab. S2** lists the details of the antibodies. 4′,6-diamidino-2-phenylindole (DAPI; catalog 28718-90-3) was purchased from Sigma-Aldrich.

**Animals**

Inbred Lewis rats (18 months old) were used. The Animal Care and Use Committee of GuangZhou Red Cross Hospital Medical College of Ji-Nan University approved all the animal experiments, which were conducted in compliance with the Guide for the Care and Use of Laboratory Animals published by the National Academy Press.

**Rat models and experimental protocol**

Myocardial infarct (MI) was induced in inbred Lewis rats by ligating the left anterior descending (LAD) coronary artery. Animals with an ejection fraction (EF)<70% and fractional shortening (FS)<35% evaluated by echocardiography after induction of MI were selected [6]. To exactly valuate the pre-existing vascular niche at the tissue, cellular, and molecular levels, ten animals were sacrificed to evaluate vessel density, Ang1, bFGF, and VEGF in the peri-infarct areas at day 1, day 7, day 14, day 21, and day 28 post-MI.

After establishment of MI model, the animals were randomly divided into three groups corresponding to FoxC1 transfection status: knockdown of FoxC1 by transfecting the infarcted hearts with vectors encoding FoxC1 siRNA (*si*FoxC1 (+) group), no-intervention, and overexpression of FoxC1 by transfecting the IHs with vectors encoding FoxC1 (*ad*FoxC1(+) group). No-intervention rats were transfected with control vectors (-). The rats that were subjected to the same surgical procedure, except for ligation of the coronary artery, received no adFoxC1 or siFoxC1, and served as the sham group (Sham). After 15 days post-induction, ten animals in each group were sacrificed to exactly valuate the FoxC1-induced vascular environment at the tissue, cellular, and molecular levels. Meanwhile, other animals received MSCs injection. The animals from the adFoxC1 and control vector groups randomly received injection of MSCs pre-treated with *ad*Oct4 or *si*Oct4 small interfering RNAs (*si*RNAs) and control siRNA duplexes. To minimize postoperative pain, 2.5% bupivacaine was sprayed at the point of incision immediately before closure, and buprenorphine hydrochloride (0.03 mg/kg) was administered intramuscularly. After the final layer of skin was closed, triple antibiotic ointment (neomycin sulfate, polymyxin B sulfate, and bacitracin zinc) was applied to the wound. Cyclosporin A (Novartis Pharma) was administered daily (5 mg/kg, i.h.) from the first day after MI until the animals were sacrificed on day 30. Finally, twenty animals were studied in each subgroup.

**Echocardiographic measurements**

Transthoracic Doppler echocardiographic studies were performed with a 7.5-MHz phased-array transducer (Acuson Sequoia 256). Two-dimensional images were obtained at the mid-papillary and apical levels. The averages of Left ventricular end-diastolic volume, internal diameter, anterior wall thickness, posterior wall thickness at diastolic phase (LVEDV, LVEDD, LVAWd, and LVPWd, respectively), and LV fractional shortening (FS) were calculated, on the basis of three successive cardiac cycles. The experienced technician was blinded to the experimental group assignment.

**Immunohistochemistry**

The peri-infarct region was paraffin-embedded and cut into serial 10-µm slices. Quantitation of vascular area was performed using the JACoP Image J plugin. Vessels were identified as round or elliptical structures with a central lumen lined by factor VIII^+^ cells and counted in 10 randomly selected fields per section, 10 sections per heart, by a pathologist who was blinded to treatment group identity. Vascularity was expressed as the number of factor VIII^+^ vessels per mm^2^ of tissue section [5]. FoxC1 expression levels in EC positive cells or ischemic hearts were determined using the JACoP Image J plugin [9]. In each case, 5 independent images from each area were analyzed from each section.

**Enzyme-linked immunosorbent assays (ELISA)**

The levels of FoxC1, Oct4, Ang-1, bFGF, HGF, VEGF, IL-6, IL-4, and TGF-β1 in the supernatant of heart tissues or cells were measured by ELISA using a duoset methodology (R&D Systems; Minneapolis, MN). Briefly, after standard procedures, the tissue-free samples were pipetted into the wells of the microtitre plates, specific horseradish peroxidase-linked polyclonal antibodies were added and immunoreactive levels of FoxC1, Oct4, Ang-1, bFGF, HGF, VEGF, IL-6, IL-4, and TGF-β1 were determined. Values below the detection limit were assumed as zero.

**Isolation and culture of microvascular endothelial cells (ECs)**

Microvascular ECs were prepared from the ventricles of Lewis rats as described previously [10]. Briefly, as shown in **Fig. S1**, rat ventricles were minced, and cells were isolated by eight subsequent trypsinization steps, with incubation at 30 °C. Microvascular ECs were attained by adding 2 mL of EC medium (Medium 199 EARLE, 2.2 g/L NaHCO3, 20% FCS, 1% penicillin/streptomycin, 1% l-glutamine, No. F0615; Biochrom AG). The cell suspension was centrifuged for 5 min (1000 rpm, 4°C) and the pellet was resuspended in EC medium. Next, the cell suspension was filtered through a 40-μm metal mesh once, and cells were disseminated into 250-mL (75 cm2) gelatin-coated cell culture flasks (Falcon, Becton Dickinson, Heidelberg, Germany) in EC medium containing 1% fibroblast growth factor.

To purify the ECs, the cells were incubated with antibodies recognizing blood vascular cell marker proteins von Willebrand factor (vWF, also known as factor VIII) polyclonal and CD31, respectively (at 5 μg/2×10^5^ cells), following the manufacturer's instructions. Magnetically positively labeled cells were isolated using a magnetic column (LS MACS separation columns; Miltenyi Biotec). The vWF^+^CD31^+^ eluate was resuspended in EC medium, and cultured in 250 mL cell culture flasks. After purification, the ECs were used in subsequent studies, as shown in **Fig. S1**.

**Hypoxic or normoxic treatment**

Cells were removed and exposed to hypoxic (37 ℃, 93% N_2_, 5% CO_2_, and 2% O_2_) oxygen levels in a water-jacketed CO_2_ incubator; the hypoxic oxygen level was maintained via regulated nitrogen injection (Forma Scientific). Cells cultured under standard conditions (21% O_2_, 5% CO_2_, and 74% N_2_) served as normoxic control cultures. The hypoxic condition was maintained throughout the performance of all subsequent analyses.

**Visualization of gene expression heatmaps**

Gene expression heatmaps were performed by Miltenyi Biotec Genomic Services as previously described [17]. Briefly, total RNA was isolated and used to synthesize double-stranded cDNA, and then biotinylated antisense complimentary RNA (cRNA) was generated through transcription using a Genechip Expression 3'-Amplification Reagent for IVT Labeling kit (Affymetrix, Inc. USA). Next, the biotinylated labeled cRNA was hybridized to the Affymetrix Rat 230 2.0 GeneChip array (P/N900470, Affymetrix Inc., USA), stained with an antistreptavidin antibody, followed by a second staining step using a streptavidin-phycoerythrin conjugate. Fluorescence was detected using the Genechip System Confocal Scanner (Hewlett-Packard), and analysis of the data from each GeneChip was conducted using the GeneChip 3.1 software produced by Affymetrix, using the default settings. Heatmaps were generated using the R add-on package pheatmat to reflect gene expression values under several conditions [7].

**Gene silencing via RNA interference**

For the short hairpin RNA (shRNA) experiments, cells were plated into 10 cm^2^ dishes at a density of 5 × 10^4^/cm^2^ culture and infected with Lenti-*Foxc1* shRNA (TL513221V, [OriGene Technologies](https://www.biocompare.com/100314-OriGene-Technologies/)), Lenti-*HIF1* shRNA (sc-45919-V, [Santa Cruz Biotechnology](https://www.biocompare.com/100404-Santa-Cruz-Biotechnology-Inc/)), Lenti-*cFos* shRNA (sc-29221-SH, [Santa Cruz Biotechnology](https://www.biocompare.com/100404-Santa-Cruz-Biotechnology-Inc/)), Lenti-*BCLF1* shRNA (RC224372L1V, [OriGene Technologies](https://www.biocompare.com/100314-OriGene-Technologies/)), Lenti-*Sp1* shRNA (C02001-7998, [GenePharma](https://www.biocompare.com/106409-GenePharma/)), Lenti-*Ang1* shRNA (TR711949, [OriGene Technologies](https://www.biocompare.com/100314-OriGene-Technologies/)), Lenti-*bFGF* shRNA (TR710311, [OriGene Technologies](https://www.biocompare.com/100314-OriGene-Technologies/)), Lenti-*HGF* shRNA (TR709834, [OriGene Technologies](https://www.biocompare.com/100314-OriGene-Technologies/)), Lenti-*VEGF* shRNA (TF711624, [OriGene Technologies](https://www.biocompare.com/100314-OriGene-Technologies/)), and Lenti-*FoxO1* shRNA, respectively. The siRNA for *FoxO1* was purchased from Dharmacon (cat. no.D003006060020, Dharmacon Inc, Shanghai, China).

**Quantitative real-time reverse transcriptase polymerase chain reaction (qRT-PCR)**

Messenger RNAs from cultured MSCs were isolated using commercial kits. Total RNA was extracted using RNA-Stat (Iso-Tex Diagnostics, Friendswood, TX, USA) according to the manufacturer’s instructions. The extracted RNA (500 ng) was converted into cDNA using Taqman Reverse Transcription Reagents (Applied Biosystems, Foster City, CA, USA). All probes and primers were designed using Express Primer 3 software developed by the Whitehead Institute for Biomedical Research (Cambridge, MA, USA). The nucleotide sequences of selected genes were obtained from GenBank, and the primer information is shown in Tab. S1. The increase in fluorescence of 6-carboxyfluorescein (6-FAM) was automatically measured during quantitative real-time polymerase chain reaction (qPCR). Cycle thresholds (CT) for the individual reactions were determined using the ABI Prism SDS 2.0 data processing software (Applied Biosystems). Relative mRNA transcript levels were quantified using the 2^-ΔΔ^ CT method, with GAPDH as an internal control.

**Western blotting**

To confirm the levels of proteins of interest, western blotting assays were performed as described previously, with modifications [10]. In brief, protein extracts (100 μg per sample) were separated using sodium dodecyl sulfate polyacrylamide gel electrophoresis (SDS-PAGE; Bio-Rad Laboratories, Hercules, CA, USA) and electrotransferred onto polyvinylidene difluoride (PVDF) membranes (GE Healthcare, Piscataway, NJ, USA). The membranes were probed with primary antibodies (**Tab. S2**) and then with labeled secondary antibodies. GAPDH and β-actin served as a positive control. The target protein levels were determined as the ratios of the target protein/GAPDH using Image-Quant software (GE Healthcare).

**Immunocytofluorescence**

Cells were fixed in 4% paraformaldehyde solution for 2 h at 4 °C and mounted on slides. Before immunostaining, the slides were pretreated with citrate buffer for antigen retrieval according to standard protocols. Cells were stained with primary antibodies, followed by incubation with FITC or TRITC-conjugated secondary antibodies. All cells were then stained with DAPI (10 µg/ml, Santa Cruz Biotechnology) in PBS for 5 min. Immunofluorescence controls were performed to assess specificity, including exclusion of the primary antibody and use of mouse, goat, and rabbit sera isotypes in place of the antibodies. Vascular differentiation was evaluated by calculating the proportion of cells that expressed both vWF and CD31. Collage I/ vimentin double positive cells could represent collagen. A pathologist who was blinded to the group identity of the slides evaluated the parameters.

**Cell culture of ECs colony number**

The ECs was resuspended in EC medium were cultured in growth medium (Medium 199, Biological Industries, Israel) supplemented with 20% heat inactivated FCS, gentamycin (50 μg/ml) and amphoterycin-B (25 μg/ml). Growth medium was replaced every 3 days, and the numbers of colonies were assessed 7 days after the initial plating. A colony was defined as a central cluster of round cells with multiple spindle-shaped cells radiating from the periphery. Colony growth was monitored, and plates fixed and stained with acetone/methanol (1:1) and Giemsa [2]. The colony numbers per well were counted and averaged in 4 wells in each experiment at ×10 magnification under an inverted microscope (Olympus, Paris, France) [10].

**Growth kinetics and replicative capacity**

At first passage, 0.5% (w/v) 3-(4,5-dimethylthiazol-2-yl)-2,5-diphenyltetrazolium bromide (MTT) in PBS was added to each well and the mixture was incubated at 37°C for 4 h. Afterwards, dimethyl-sulfoxide was added to the mixture and the mixture was incubated with gentle shaking for 10 min. Cell viability was determined by the absorbance at 570 nm using a micro-plate reader [10]. At each subsequent passage, cells were enumerated for calculation of a growth kinetic curve. The percent of the viable cells was calculated as the ratio of unstained cells to total cells, and the numbers of stained and unstained cells were counted over three randomly selected high-power fields.

**Tube formation and angiogenesis of ECs *in vitro***

Capillary tube formation of ECs was evaluated as follows. 24-well 18 mm tissue culture dishes were coated with Matrigel basement membrane matrix (300 mL per well; Becton Dickinson Labware) at 4°C and allowed to polymerize at 37°C for at least 30 min. ECs (2×10^5^ cells per well) were grown in a final volume of 0.30 mL culture medium containing 150 mL M199 (GibcoBRL) and 150 mL CM. After 6 h incubation, tube formation was observed through a reverted, phase-contrast photomicroscope photographed and counted. The number of tube formations was measured by counting the number of tube like structures formed by connected endothelial cells in five randomly selected 9.7mm^2^ microscopic fields. The assay was performed in triplicate.

After induction of vascular differentiation, the collected cells were washed with PBS and analyzed using immunocytofluoroscence and flow cytometry (Becton Dickinson). The cells (2 × 10^5^/ml) were then incubated with 4 μl of antibodies against factor VIII (vWF) and α-SMA or CD31. Vascular differentiation of ECs was evaluated by calculating the proportion of cells that expressed both vWF and α-SMA.

**Cell proliferation and apoptosis analysis**

The cell viability assay was measured using a cell counting kit-8 (CCK-8, Sigma) according to the manufacturer’s protocol. Cells were seeded in 96-well plates with 5,000 cells per well. After treatment, 10 μl/well of CCK-8 solution was added into each well and incubated for 4 h. The absorbance was measured at 450 nm using a microplate reader (Bio-Rad, Hercules, CA, USA) [12]. Cell proliferation was assessed using fluorescence staining and FACS for proliferation markers (5-bromodeoxyuridine (BrdU) and Ki67) following the company's protocol. Photomicrographs were taken at ×20 magnification in four randomly selected fields in each well. For BrdU incorporation studies, BrdU labeling reagent (Amersham) was diluted 1:1000 in medium to a final concentration of 10 μM BrdU, and sterilized through a 0.22 μm filter. 2 ml of staining solution were added to each well of a 6-well plate, followed by incubation for 12, 24, 36, or 48 hours as indicated. After washing, fixation, and incubation, 20 μl anti-BrdU antibody (Becton-Dickinson) was added, followed by 30 minute incubation at room temperature in the dark. Flow cytometry was done on a FACScan (Becton-Dickinson) and results were analyzed using FlowJo software (TreeStar) [3]. Live cells were analysed by flow cytometry to identify the population in S-phase on the basis of the level of DNA fluorescence [1].

Apoptotic cell death under hypoxic conditions was evaluated through annexin V (Roche Diagnostic, Indianapolis, IN) and propidium iodide (PI) staining using a FACScan flow cytometer and Cell Quest software (Becton Dickinson Immunocytometry Systems, San Jose, CA) and TUNEL assay. At the time point of culture observation, 2×10^6^ cells were resuspended in PBS and homogenized in buffer containing proteinase K and RNase. After 15 minutes of incubation at 37°C, NaI solution was added. Cell lysates were incubated at 50°C for 30 minutes and isopropanol was added. DNA was precipitated by centrifugation and washed by 70% ethanol. DNA (8 μg) was then analyzed using 1.2% agarose gel electrophoresis and visualized under a UV (302 nm) transiluminator. Annexin V staining was performed with a commercially available kit (Roche) according to the manufacturer’s protocols and annexin V positive cells were counted by FACS. Apoptotic cells were also evaluated by TUNEL assay with an ApopTag kit (17-141, Upstate). Cell sections were examined microscopically. Four fields each were selected in each section. The percentage of apoptotic cells was termed the apoptotic index.

**Acetylated low-density lipoprotein (acLDL) uptake and *Ulex europaeus* agglutinin-1 (UEA-1) binding test**

After the induction of MSC differentiation into blood vascular cells, the cells were characterized as adherent cells double-positive for DiI (1,1'-dioctadecyl- 3,3,3',3'-tetramethylindocarbocyanine perchlorate)-acLDL (Biomedical Technologies, USA) uptake and fluorescein isothiocyanate (FITC)-UEA-1 (Sigma, USA) binding. After induction in culture, the adherent cells were incubated with 10 μg/ml DiI-acLDL for 12 h at 37 °C, fixed with 4% paraformaldehyde (Sigma-Aldrich), and then stained with 10 μg/ml FITC-UEA-1 for 3 h at room temperature. Nuclei of the cells were counterstained with 1 μg/ml DAPI for 15 min at room temperature. The cells were then visualized at 200 × magnification using a laser scanning confocal microscope (LSM710; Carl Zeiss, Germany) [15].

**Isolation, expansion, and purification of mesenchymal stem cells (MSCs)**

MSCs were isolated from rat bone marrow, and cultured via the adherent culture method, as described previously [13, 14]. Briefly, mononuclear cells (MNCs) were cloned via limited dilution, and expanded in MSC complete medium (Iscove’s modified Dulbecco’s medium: IMDM with 20% fetal bovine serum (FBS), 2 mM L-glutamine, penicillin [100 U/ml], and streptomycin [100 µg/ml]). When the cells reached 80% confluence, they were collected and their purity and characteristics were determined using fluorescence activated cell sorting (FACS). The characteristics of the MSCs were confirmed using antibodies recognizing CD34, CD44, CD45, CD71, CD90, CD133, CD147, SH2, and SH3. Mouse IgG1, IgG2a, and IgG2b (Becton Dickinson) were used as isotype controls, and marker expression was evaluated using FACS as mentioned above. Cell viability was evaluated using a propidium iodide exclusion assay and flow cytometry. FACS showed that 95.6-98.1% of the MSCs expressed CD44, CD71, CD90, CD147, SH2, and SH3, whereas they expressed less than 2% of CD34, CD45, and CD133 (**Fig. S2**).

**Establishment of hypoxic co-culture model of ECs and MSCs**

We used Millicell Culture Plate Inserts to establish the hypoxic co-culture model of ECs and MSCs. Hypoxic culture was carried out in a hypoxic incubator at 37 ℃, 93% N_2_, 5% CO_2_, and 2% O_2_. The transwells containing MSCs were transferred to the plates where ECs were previously cultured. The permeable PET membrane of 1 μm pore size avoided MSCs to cross from the top to the lower compartment, allowing however the exchange of soluble factors between ECs and MSCs. The co-cultures were maintained in 5 ml of IMDM in the above-mentioned hypoxic environment. MSCs and ECs were seeded into the upper layer and the bottom layer of the cell culture inserts at a 1:3 ratio, and the medium was replaced with the optimized medium included in the Angiogenesis Kit (KZ-1500; Kurabo Industries), containing 2% FBS that is free of VEGF, bFGF and hypoxia-inducible factor (HIF) [8, 10]. The experimental setup and respective conditions are schematically presented in**[Fig](https://www.frontiersin.org/articles/10.3389/fimmu.2019.01508/full#F1). S1**.

**Cell treatments and groups**

As described in **Fig. S1**, the purified MSCs were divided into three groups: First, coculture (MSCs+ECs) was performed to compare the effects of FoxC1 on the growth, proliferation, and apoptosis of MSCs with that of monoculture under hypoxic condition; second, MSCs were added with *ad*Oct4 or *si*Oct4, and cocultured with ECs pre-treated by *Foxc1* transfection (*ad*Foxc1) or not (*-*) under hypoxia to analyze their long-term survival and mesenchymal-to-endothelial transition (MEndoT); third, MSCs were transfected with *ad*Oct4, *s*iOct4, or not, and injected into the infarcted hearts to investigate their survival and MEndoT *in vivo*.

In coculture, ECs were subjected to hypoxic coculture to analyze the effects of FoxC1-mediated hypoxic microenvironment on the survival, MEndoT, and angiogenesis of MSCs. Coculture was performed in the absence (-) or presence of ECs transfection of FoxC1 overexpression (*ad*Foxc1), FoxC1 silencing (*si*Foxc1), or not (-). ECs (5 × 10^4^ cells/0.5 ml well) were plated in triplicate in 24-well plates and pre-cultured overnight in Dulbecco’s modified Eagle’s medium (DMEM). The medium was removed and the ECs were cocultured with MSCs under hypoxic conditions (2% O_2_). MSC population doubling, proliferation, and apoptosis were assessed at 108 h of culture.

To investigate the role of octamer binding protein 4 (Oct4) in the survival and self-renewal of MSCs under hypoxic conditions, Oct4 was overexpressed or inhibited by transfecting with *ad*Oct4 or *si*Oct4 at 72h post-hypoxic coculture with ECs alone. Blank vector served as control. Then, these cells were co-cultured with ECs pre-treated with *ad*FoxC1 or not. At the end of each required observation endpoint, MSCs were collected and subjected to molecular biology examination to analyze their angiogenesis, MEndoT, and the expressions of angiogenic cytokines.

**FoxC1 and Oct4 transfection**

Transfection of the adenoviral vector encoding the recombinant rat FoxC1 or Oct4 gene under the control of a cytomegalovirus promoter (*ad*Foxc1 or *ad*Oct4) and Ad.Null (Ctrl) was performed using nucleoporation (Nucleofector, Amaza) according to the manufacturer’s protocol. The oligonucleotides and their complementary versions were synthesized by USEN Tech (Shanghai, China), annealed, and ligated into the vectors. For the expression of FoxC1 or Oct4, the full length sequence of its protein coding sequence was amplified by PCR using primers, and then ligated into the vectors. Transfection efficiency was approximately 70%, as determined using green fluorescent protein, and maximal levels of protein expression were observed between 24 and 48 hours. To knock down Oct4 or FoxC1 expression, transfection was performed using small interfering RNA (*si*RNA) targeted to FoxC1 or Oct4 as described previously [16]. In brief, to formulate the lipid-siRNA complex, the siRNA (Origene Technologies) and Lipofectamine 2000 (Invitrogen) were diluted separately in Opti-MEM (Invitrogen).

**Angiogenesis Assay**

To determine whether Oct4 affects blood vascular growth factors and cytokines, MSCs were analyzed for the presence or absence of Oct4 using a RayBio Quantibody Rat Angiogenesis Array 100 kit (QAR-ANG-100, RayBiotech, GA, USA). The assay was performed as instructed by the manufacturer and as described previously [9]. In brief, 100 µl of Blocking Buffer was added into each well and incubated at room temperature for 60 min. After removing the blocking buffer, 30 µg of samples or serial diluted cytokine standards were added to each well containing 70 µl of Sample Diluent. After overnight incubation at 4 ℃, the samples were decanted and washed three times with wash buffer I at room temperature with gentle shaking. The cells were washed twice with wash buffer II and then 70 µl of diluted Detection Antibody was added to each well. The plates were incubated at room temperature for 1 h and washed as above. The signal of array glass chip was scanned using Axon GenePix (GenePix 4000B, Axon Instruments, USA). Data were analyzed using GenePix Pro 6.0.

**FACS of MSC vascularization and fibrosis**

After induction of vascular differentiation, the collected cells were washed with PBS and analyzed using flow cytometry (Becton Dickinson) and sorted by FACS. The cells (2 × 10^5^/ml) were then incubated with 4 μl of antibodies against vWF, CD31, collage I and vimentin. Data were acquired on the FACSCanto II (BD Biosciences) and analyzed with FCS Express (De Novo Software). Vascular differentiation was considered as percent of vWF/CD31 double-positive staining. Collagen I and vimentin expressions were analyzed as fibrosis makers.

**EGFP labelling**

At 24 h after transfection with the Oct4, siOct4, or control siRNA vectors, cells were co-transfected with a lentiviral vector containing enhanced GFP (EGFP) cDNA, as described previously [16]. More than 70% of MSCs were EGFP-positive, as determined by flow cytometry. Subsequently, these cells were randomly injected into the FoxC1-induced ischemic vascular niches.

**MPO and ROS detection**

30 days after cell transplantation, the animals were killed with i.p. injection of overdose barbiturate (150 mg/kg) after the echocardiography examinations, weighed, and their hearts were removed. Ten hearts were randomly selected for subsequent cell collection. The other hearts were divided into three transverse slices from the base, mid-region, and apex. The base slices were homogenized in saline (per 100 mg tissue in 0.9 ml saline). After 15-min centrifugation at 3000 ×*g*, the supernatant was collected and stored at -70°C until used. Myocardium myeloperoxidase (MPO) and reaction oxygen species (ROS) were detected according to the manufacturer’s instructions (A044, A018, NJBI). The peroxide-sensitive fluorescent probe 2′,7′-dichlorodihydrofluorescein diacetate (Molecular Probes) was used to assess the generation of intracellular ROS as described previously [15]. This compound is converted by intracellular esterases to 2′,7′-dichlorodihydrofluorescein (DCF), which is then oxidized by hydrogen peroxide to highly fluorescent DCF. Differential interference contrast images were obtained simultaneously using an Olympus inverted microscope with ×40 Aplanfluo objective and an Olympus fluoview confocal laser-scanning attachment. The DCF fluorescence was measured with an excitation wave length of 488 nm of light, and its emission was detected using a 510–550-nm bandpass filter. The change in MPO absorbance was measured at 460 nm using a spectrophotometer. One unit of MPO was defined as the quantity of enzyme required to hydrolyze peroxide at a rate of 1 mmol/min at 25°C.

**Histology and immunohistofluorescence**

Hematoxylin/eosin (H&E), masson trichrome, or immunoflfluoroscence staining was performed to evaluate infarct size, collagen content, or vessel density, respectively. Two 5-μm sections from each paraffin-embedded slice were obtained and were stained with H&E staining. H&E staining was performed on each LV cross section to examine myocardial injury and inflammatory responses. MI size was evaluated by 2,3,5-triphenyltetrazolium chloride triazole (TTC) staining [11]. The middle portion of the left ventricles was immersed in 0.09 mol/l PBS (pH 7.4) containing 1.0% TTC (Sigma-Aldrich) for 20 min at 37°C. Infarct area was calculated as the ratio between scar area and the area of the whole-LV section and expressed in percentages [4]. In order to assess the fate of engrafted cells in the peri-infarct and infarct areas, the number of engrafted cells was counted under flfluorescence microscope on a series of cryostat left ventricular apical tissue sections. The number of EGFP^+^ cells was evaluated by counting ten randomly selected high-power fields.

Immunofluorescent staining on frozen sections (7μm) was performed using primary antibodies to CD31, CD68, and Factor VIII, followed by incubation with FITC or TRITC-conjugated secondary antisera. FoxC1 expression levels in cardiac endothelial cells were determined using the JACoP Image J plugin and expressed as the percent proportion of the cells double positively stained with FoxC1 and CD31 to the total positive- CD31 staining cells. The amount of MEndoT was derived by counting the number of CD31 positive cells per high-fold field. Quantitation of vascular area derived from endothelial cells was performed using the JACoP Image J plugin [9]. The ratios of MSC transformation into inflammatory cells were expressed as the percent proportion of the cells double positively stained with EGFP and CD68 to the total positive-EGFP staining cells. The number of vessels was expressed as the number of factor VIII^+^ endothelial cells per high-fold field. Immunofluoroscence controls were performed to assess specificity, including exclusion of primary antibody and use of mouse, goat, and rabbit sera isotype in place of the antibodies. A pathologist who was blinded to the group identity of the slides evaluated the parameters.

**FACS of Engrafed MSCs**

Cells were collected from the left ventricles of ten randomly selected hearts per experimental group as previously described [16]. Briefly, the heart was excised and perfused retrograde with Ca^2+^-free perfusion buffer. The left ventricle was minced in collagenase, and the solution was filtered through a nylon mesh. The collected cells were washed with PBS and analyzed with a flow cytometry apparatus (Becton Dickinson, Mountain View, CA, USA). Engraftment was evaluated by determining the proportion of cells that expressed EGFP relative to all isolated ventricular cells.

**Statistical analysis**

Data are presented as the mean ± standard error of the mean (SEM). Discrete variables are presented as frequency and proportion. By performing normality test (Shapiro-Wilk test) and homogeneity test of variance, the data that satisfy normal distribution and equal variance assumptions were used for one-way ANOVA analysis of these variables. When the data were conferred for normal distribution but non-homogeneity of variance, Welch ANOVA analyses were performed. Comparisons were performed using the χ^2^ or Fisher’s exact test for discrete variables A 95% confidence interval (CI) (p < 0.05) was considered significant.

**References**

1. Arai Y, Pulvers JN, Haffner C, Schilling B, Nüsslein I, Calegari F, Huttner WB (2011) Neural stem and progenitor cells shorten S-phase on commitment to neuron production. Nat Commun 2:154 doi: 10.1038/ncomms1155
2. Bruna A, Greenwood W, Le Quesne J, Teschendorff A, Miranda-Saavedra D, Rueda OM, Sandoval JL, Vidakovic AT, Saadi A, Pharoah P, Stingl J, Caldas C (2012) TGFβ induces the formation of tumour-initiating cells in claudin^low^ breast cancer. Nat Commun 3:1055 doi: 10.1038/ncomms2039
3. Cormier SA, Mello MA, Kappen C (2003) Normal proliferation and differentiation of Hoxc-8 transgenic chondrocytes in vitro. BMC Dev Biol 3: 4 doi: 10.1186/1471-213x-3-4

4. Fiordaliso F, Chimenti S, Staszewsky L, Bai A, Carlo E, Cuccovillo I, Doni M, Mengozzi M, Tonelli R, Ghezzi P, Coleman T, Brines M, Cerami A, Latini R (2005) A nonerythropoietic derivative of erythropoietin protects the myocardium from ischemia-reperfusion injury. Proc Natl Acad Sci U S A 102:2046-2051. doi: 10.1073/pnas.0409329102

5. Hoskin PJ, Sibtain A, Daley FM, Wilson GD (2003) GLUT1 and CAIX as intrinsic markers of hypoxia in bladder cancer: relationship with vascularity and proliferation as predictors of outcome of ARCON. Br J Cancer 89: 1290-1297 doi: 10.1038/sj.bjc.6601260

6. Ma J, Ge J, Zhang S, Sun A, Shen J, Chen L, Wang K, Zou Y (2005) Time course of myocardial stromal cell-derived factor 1 expression and beneficial effects of intravenously administered bone marrow stem cells in rats with experimental myocardial infarction. Basic Res Cardiol 100:217-223 doi: 10.1007/s00395-005-0521-z

7. Shen Y, Zhang R, Xu L, Wan Q, Zhu J, Gu J, Huang Z, Ma W, Shen M, Ding F, Sun H (2019) Microarray analysis of gene expression provides new insights into denervation-induced skeletal muscle atrophy. Front Physiol 10:1298. doi: 10.3389/fphys.2019.01298 6

8. Silva AJ, Ferreira JR, Cunha C, Corte-Real JV, Bessa-Gonçalves M, Barbosa MA, Santos SG, Gonçalves RM (2019) Macrophages Down-Regulate Gene Expression of Intervertebral Disc Degenerative Markers Under a Pro-inflammatory Microenvironment. Front Immunol 10:1508 doi: 10.3389/fimmu.2019.01508

9. Ubil E, Duan J, Pillai IC, Rosa-Garrido M, Wu Y, Bargiacchi F, Lu Y, Stanbouly S, Huang J, Rojas M, Vondriska TM, Stefani E, Deb A (2014) Mesenchymal-endothelial transition contributes to cardiac neovascularization. Nature 514:585-90 doi: 10.1038/nature13839

10. Wang JH, Zhao L, Pan X, Chen NN, Chen J, Gong QL, Su F, Yan J, Zhang Y, Zhang SH (2016) Hypoxia-stimulated cardiac fibroblast production of IL-6 promotes myocardial fibrosis via the TGF-β1 signaling pathway. Lab Invest 96:839-852. doi: 10.1038/labinvest.2016.65

11. Xu L, Zhou J, Liu J, Liu Y, Wang L, Jiang R, Diao Z, Yan G, Pèault B, Sun H, Ding L (2017) Different angiogenic potentials of mesenchymal stem cells derived from umbilical artery, umbilical vein, and wharton's jelly. Stem Cells Int 2017:3175748 doi: 10.1155/2017/3175748

12. Yin D, Fu C, Sun D (2018) Silence of lncRNA UCA1 represses the growth and tube formation of human microvascular endothelial cells through miR-195. Cell Physiol Biochem 49:1499-1511 doi: 10.1159/000493454

13. Zhang S, Jia Z, Ge J, Gong L, Ma Y, Li T, Guo J, Chen P, Hu Q, Zhang P, Liu Y, Li Z, Ma K, Li L, Zhou C (2005) Purified human bone marrow multipotent mesenchymal stem cells regenerate infarcted myocardium in experimental rats. Cell Transplant 14:787-798. doi: 10.3727/000000005783982558 46

14. Zhang S, Lu S, Ge J, Guo J, Chen P, Li T, Zhang P, Jia Z, Ma K, Liu Y, Zhou C, Li L (2005) Increased heme oxygenase-1 expression in infarcted rat hearts following human bone marrow mesenchymal cell transplantation. Microvasc Res 69:64-70. doi: 10.1016/j.mvr.2005.01.004

15. Zhang S, Ma X, Guo J, Yao K, Wang C, Dong Z, Zhu H, Fan F, Huang Z, Yang X, Qian J, Zou Y, Sun A, Ge J (2017) Bone marrow CD34+ cell subset under induction of moderate stiffness of extracellular matrix after myocardial infarction facilitated endothelial lineage commitment in vitro. Stem Cell Res Ther 8:280. doi: 10.1186/s13287-017-0732-x

16. Zhang S, Zhao L, Wang J, Chen N, Yan J, Pan X (2017) HIF-2α and Oct4 have synergistic effects on survival and myocardial repair of very small embryonic-like mesenchymal stem cells in infarcted hearts. Cell Death Dis 8:e2548. doi: 10.1038/cddis.2016.480

17. Zhu P, Wang Y, He L, Huang G, Du Y, Zhang G, Yan X, Xia P, Ye B, Wang S, Hao L, Wu J, Fan Z (2015) ZIC2-dependent OCT4 activation drives self-renewal of human liver cancer stem cells. J Clin Invest 125:3795-808 doi: 10.1172/JCI81979

**Supplementary Figure and Table Legends**

**Supplementary Fig. S1. Flow chart of cell preparation, gene transfection, coculture, transplantation, and grouping.**

**Supplementary Fig. S2. Surface marker expression of rat MSCs.** (**A**) Flow cytometry analysis of the immunophenotypic surface profiles for SH2, SH3, CD44, CD71, CD90, CD147, CD34, CD45, and CD133 of cultured MSCs. Histograms represent the counts of cells incubated with the relevant antibody. The logarithm on the x-axis represents the intensity of the fluorescent signal, and the number of cells are shown on the y-axis. Third passage cultured MSCs were positive for the markers SH2, SH3, CD44, CD71, CD90, and CD147; but negative for CD34, CD45, and CD133. (**B**) Graph representing data based on three independent experiments.

**Supplementary Fig. S3**.**Serial Changes of blood vascular density and pro-angiogenic cytokines in the ischemic areas after MI induction**. (**A**) Representative immunohistochemical staining images of factor VIII under lower magnifification (×10, upper, Scale bars: 100 µm.) and higher magnifification (×40, lower, Scale bars: 50 µm.) by staining vessels at days 1, 7, 14, 21, and 28 post-MI in the infarct and peri-infarct area from MI hearts. Factor VIII was stained as brown in the cytoplasm of blood vascular cells. (**B**) The temporal changes of blood vascular density were determined by the number of factor VIII positive-staining vessels per mm^2^ under high-power fifield view by immunohistochemistry after MI. (**C**-**E**) ELISA assay of difference in Ang1 (**C**), bFGF (**D**), and VEGF (**E**) at days 1, 7, 14, 21, and 28 post-MI in the infarcted hearts. All data represent means±s.e.m. *p*<0.05: ^*^vs 1d, ^†^vs 7d, ^‡^vs 14d, and ^§^vs 21d post-MI in each group (n=10 in each group). Data are representative of at least 3 independent experiments. Welch ANOVA analyses were performed for statistical analysis.

**Supplementary Fig. S4. FoxC1 deficiency impairs self-renewal of ECs under hypoxia**. (**A**) FoxC1 was knocked down in ECs. FoxC1-silenced ECs and control cells were cultured for clone-formation assays under normal or hypoxic conditions. FoxC1-silenced ECs exhibited significantly lower clonogenic potential than all other cells in all conditions; cells originated from human adult kidney from three different patients. Scale bars = 100 μm. (**B**) Top 10 transcription factors, which involved in the maintenance of blood vascular ECs self-renewal, survival, and angiogenesis, were depleted in ECs in hypoxic or normoxic cultures.Their clone formation was tested via *in vitro* assays. All data are the means ± SEM. *p* < 0.05: * *vs*. CON, ^†^ *vs*. Normoxia (*n* = 10 per group). One-way ANOVA analysis was used for statistical analysis.

**Supplementary Fig. S5. Oct4 enhances MSC-mediated amelioration of MI pathology.** (**A**) Representative high-magnification images of H&E staining. Scale bars = 50 μm. (**B**) Sections of hearts harvested at 30 days following MSC therapy were stained for the monocyte/macrophage marker CD68 (red). The nuclei were stained blue using DAPI. Scale bars=25 μm. Note that CD68 showed the smallest expression level in the EGFP labeled MSCs (green) who were transfected with *ad*Oct4 and transplanted into the *ad*FoxC1 ischemic hearts. (**C**) Masson’s trichrome staining (none of the infarcted myocardium was stained red; pale region, infarcted myocardium; bright red, viable myocardium; bright blue, fifibrosis) at 30 days post-MI. Scale bars = 50 μm. (**D**-**G**) Quantitative analysis of MPO (**D**), ROS (**E**), the percentage of CD68 cells in the EGFP labeled MSCs (**F**), and viable myocardium (**G**) 30 days after cell therapy. All graphs show means ± SEM. *p* < 0.05: in the ischemic hearts without FoxC1 transfection, ^*^ *vs* MSC therapy alone, ^†^ *vs*. Transplantation of *ad*Oct4 transfected MSCs, ^‡^ *vs*. Transplantation of *si*Oct4 transfected MSCs; in the adFoxC1 hearts, ^§^ *vs* MSC therapy alone, ^||^ *vs*. Transplantation of *ad*Oct4 transfected MSCs (*n* = 10 per group). Welch ANOVA analyses were performed in **Fig. S5D-F**, and one-way ANOVA analysis was used in **Fig. S5G**.

**Supplementary Fig. S6. ELISA assay of difference in FoxC1 (A), Oct4 (B), Ang1 (C), bFGF (D), VEGF (E), IL-6 (F), TGF-β1 (G), and IL-4 (H) at day 30 post-MI in various groups.** All graphs show means ± SEM. *p* < 0.05: in the ischemic hearts without FoxC1 transfection, ^*^ *vs* MSC therapy alone, ^†^ *vs*. Transplantation of *ad*Oct4 transfected MSCs, ^‡^ *vs*. Transplantation of *si*Oct4 transfected MSCs; in the adFoxC1 hearts, ^§^ *vs* MSC therapy alone, ^||^ *vs*. Transplantation of *ad*Oct4 transfected MSCs (*n* = 10 per group). Welch ANOVA analyses were used for statistical analysis.

**Supplementary Table S1. Primers for qRT-PCR of rat tissues.**

**Supplementary Table S2. The antibodies for fluorescence activated cell sorting (FACS), western blot (WB), enzyme linked immunosorbent assay (ELISA), immunofluoroscence (IF), and immunohistochemistry (IHC)**
